# Pseudo partition-encoded simultaneous multislab (pPRISM) for rapid, navigator-free submillimeter diffusion MRI with reduced slab-boundary signal loss

**DOI:** 10.1162/imag_a_00417

**Published:** 2025-01-03

**Authors:** Wei-Tang Chang, Congyu Liao, Hong-Hsi Lee

**Affiliations:** Biomedical Research Imaging Center, University of North Carolina at Chapel Hill, Chapel Hill, NC, United States; Department of Radiology, University of North Carolina at Chapel Hill, Chapel Hill, NC, United States; Department of Radiology, Stanford University, Stanford, CA, United States; Department of Electrical Engineering, Stanford University, Stanford, CA, United States; Athinoula A. Martinos Center for Biomedical Imaging, Massachusetts General Hospital, Charlestown, MA, United States; Department of Radiology, Harvard Medical School, Boston, MA, United States

**Keywords:** SMS, multislab, multi-shot dMRI, ultrahigh resolution dMRI, pseudo slab, PRISM

## Abstract

The primary aim of this study is to address the challenges in submillimeter diffusion magnetic resonance imaging (dMRI), such as prolonged acquisition time, low signal-to-noise ratio (SNR), and signal attenuation at slab boundary. We introduce a novel 3D Fourier encoding mechanism, PRISM (Partition-encoded Simultaneous Multislab), and a new concept termed “pseudo slab.” The PRISM method allows simultaneous inter-slab and intra-slab Fourier encoding solely using the slice gradient, eliminating the need for RF encoding. The pseudo slab concept not only minimizes inter-slab signal leakage and Gibbs truncation artifacts, but also enables phase scheduling onto intra-slab slices, thus eliminating the need for a phase navigator and time-varying gradient such as variable-rate selective excitation (VERSE). Integrating the pseudo slab with PRISM, the resulting pseudo PRISM (pPRISM) technique achieved rapid acquisition of dMRI with 0.86-mm isotropic resolution and an effective TR of 12 s (TR of 2.4 s per shot). Compared to Generalized Slice Dithered Enhanced Resolution with Simultaneous Multislice (gSlider-SMS), the shortened acquisition time improved the SNR efficiency without aggravating the signal attenuation at slab boundaries. The robustness of pPRISM against field inhomogeneity was also supported by Bloch simulation and empirical data. Furthermore, dMRI was successfully achieved with a 0.76-mm isotropic resolution, an effective TR of 15 s, and b-values of up to 2500 s/mm^2^. The ultrahigh-resolution results of the proposed pPRISM method demonstrated the anticipated dark bands of fractional anisotropy (FA) at gray-white matter boundaries and yielded more plausible tractography results. Our pPRISM framework paves the way for acquiring ultrahigh-resolution dMRI in clinically feasible times, advancing microstructural research.

## 1 Introduction

The advantage of submillimeter diffusion magnetic resonance imaging (dMRI) has been clearly demonstrated in prior studies (Assaf, 2019;Gulban et al., 2018;McNab et al., 2013;Miller et al., 2011). However, the primary challenges associated with submillimeter dMRI are its prolonged acquisition time and inherently low signal-to-noise ratio (SNR). Over the last decade, simultaneous multislice (SMS) imaging using blipped-controlled aliasing (blipped-CAIPI SMS) has emerged as a predominant method to reduce acquisition time (Setsompop et al., 2012). While the SMS method significantly decreases acquisition time, the low SNR remains an issue hindering our ability to achieve submillimeter dMRI.

Increasing both excitation volume per shot and number of shots are common strategies to enhance the SNR. This concept is embodied in two different strategies—3D multislab dMRI and radio-frequency-encoded (RF-encoded) multishot dMRI. The 3D multislab dMRI sequentially excites thick slabs during each repetition time (TR), with Fourier encoding executed along the slab direction over multiple TRs (Engström & Skare, 2013;Frank et al., 2010;Frost et al., 2014). Nevertheless, this approach comes with certain limitations. For instance, imperfect slab-selective profiles may produce slab boundary artifacts. Additionally, the use of Fourier encoding makes it difficult to self-navigate the phase variation across shots when the encoding frequency is high. Various solutions have been put forth to overcome these challenges. Notably, nonlinear inversion for slab profile encoding was introduced to alleviate slab boundary artifacts, although this requires significant computational resources (Van et al., 2015;Wu et al., 2016). Furthermore, the 2D navigator has been employed to track the phase variation in SMSlab (Bruce et al., 2017). Yet, incorporating a 2D navigator escalates both acquisition time and the specific absorption rate (SAR). Separately, Gibbs artifacts can appear with limited slab encoding numbers (Block et al., 2008). While increasing the slab-encoding shots can mitigate these artifacts, it consequently prolongs the total acquisition time if the TR per shot remains unchanged to ensure adequate longitudinal magnetization recovery.

Compared to 3D multislab techniques, the RF-encoded multishot method, specifically the Generalized Slice Dithered Enhanced Resolution with Simultaneous Multislice (gSlider-SMS) (Setsompop et al., 2018), enables the simultaneous acquisition of multiple thin slabs. Each of these slabs comprises several thin slices that are RF-encoded in a manner analogous to Hadamard encoding (Glover & Chang, 2012). The gSlider method, by virtue of its RF-encoding, presents two distinct advantages over 3D multislab techniques. Firstly, it provides a high SNR per shot, facilitating phase self-navigation. Secondly, it avoids Gibbs truncation artifacts, even when the number of slices within a slab is small. However, the gSlider-SMS approach also presents technical challenges, such as elevated RF peak amplitude and the emergence of slab boundary artifacts. Although the RF peak amplitude can be reduced by variable-rate selective excitation (VERSE) (Hargreaves et al., 2004), it renders the gSlider imaging sensitive to B1 and B0 inhomogeneity. Additionally, to alleviate the signal attenuation at slab boundaries, a relatively long TR (≥3.5 s) is necessary, which may adversely impact SNR efficiency.

As the 3D muiltislab approach enhances the SNR and SMS imaging accelerates the scan, the combination of these two methods, namely the simultaneous multi-slab (SMSlab) method, holds the promise of rapid acquisition while preserving the SNR. However, a 3D k-space description for SMSlab has yet to be formulated. Such a 3D k-space description will serve as an important reference, informing the design of sub-sampling trajectory. While both the multi-slab and SMS acquisitions can be described by 3D Fourier encoding framework (Zahneisen et al., 2013), a dedicated 3D k-space representation for SMSlab is still lacking. The difficulty of describing SMSlab in 3D k-space is that the inter-slab and intra-slab dimensions are represented by the same physical z-gradient axis. When the slice-encoding gradient is applied, it will contribute to both the inter-slab and intra-slab encoding, complicating their distinction. As of now, 3D encoding for SMSlab requires the use of both RF phase modulation and gradient encoding (Dai et al., 2019). This reliance on RF phase modulation for 3D encoding precludes the application of RF phase scrambling, a method designed to decrease the RF peak amplitude (Wong, 2012).

In this study, we will address the issues of slab-boundary attenuation and peak RF amplitude by introducing a novel 3D Fourier encoding mechanism for SMSlab that does not require RF phase modulation. We also present a new concept termed “pseudo slab”. Our 3D Fourier encoding framework for SMSlab is denoted as PRISM, standing for Partition-encoded Simultaneous Multislab. PRISM encoding performs, for the first time, simultaneous inter-slab and intra-slab Fourier encoding solely utilizing the slice gradient. Comprehensive mathematical derivations and illustrative figures elucidating PRISM are provided in this study. Moreover, our introduced concept of pseudo slab effectively mitigates slab boundary artifacts, Gibbs truncation artifacts, and reduces the peak RF amplitude. By integrating the pseudo slab with PRISM, our innovative framework, pseudo PRISM (pPRISM), enables the acquisition of submillimeter-resolution dMRI, devoid of slab boundary artifacts and Gibbs truncation artifacts, without the need for an additional phase navigator. Additionally, the decrease in peak RF amplitude obviates the need for the pPRISM method to utilize time-varying gradient VERSE, thereby enhancing robustness against B_0_field inhomogeneity. Employing the pPRISM method, we have successfully acquired dMRI with an isotropic resolution of 0.76 mm, a b-value up to 2500 s/mm^2^, and 128 diffusion directions in approximately 33 minutes. Our ultra-high-resolution results not only exhibited the anticipated dark bands of fractional anisotropy (FA) at the boundaries between gray and white matters but also yielded more plausible tractography results compared to standard-resolution imaging.

## 2 Theory

### 2.1 The principles of PRISM

The PRISM method is proposed to perform both inter-slab and intra-slab Fourier encoding simultaneously. Assuming the number of SMSlab is M and each slab is to be partitioned into n_p_slices, the M slabs will be partitioned into M × n_p_slices in total. The ultimate goal of the Fourier encoding for SMSlab is to form M × n_p_Fourier bases that are orthogonal to each other. An illustrative example is presented inFigure 1a. For instance, if the SMS factor is 3 and the number of slab Partition Encoding (PAE) is also 3, then a total of 9 Fourier bases are required. As shown inFigure 1b, Fourier encoding for SMSlabs is accomplished through the 1D convolution of partition encoding patterns with SMS encoding patterns along k_z_axis in k-space. Let FOV_z_denotes the size of field-of-view (FOV) in z direction. In the case of SMS encoding, the spacing between adjacent k_x_-k_y_planes can be q/FOV_z_as long as q and the SMS factor are coprime, meaning the greatest common divisor (gcd) of SMS and q must be 1, that is, gcd(SMS, q) = 1. For example, as depicted inFigure 1c, if the SMS factor is 3, permissible values for q could be 1, 2, or 4, but not 3. PRISM encoding patterns corresponding to q of 1, 2, and 4 are displayed in the left, middle, and right panels ofFigure 1c, respectively. The formal mathematical proof establishing the orthogonality of PRISM encoding can be found in theSupplementary Materials.

**Fig. 1.**
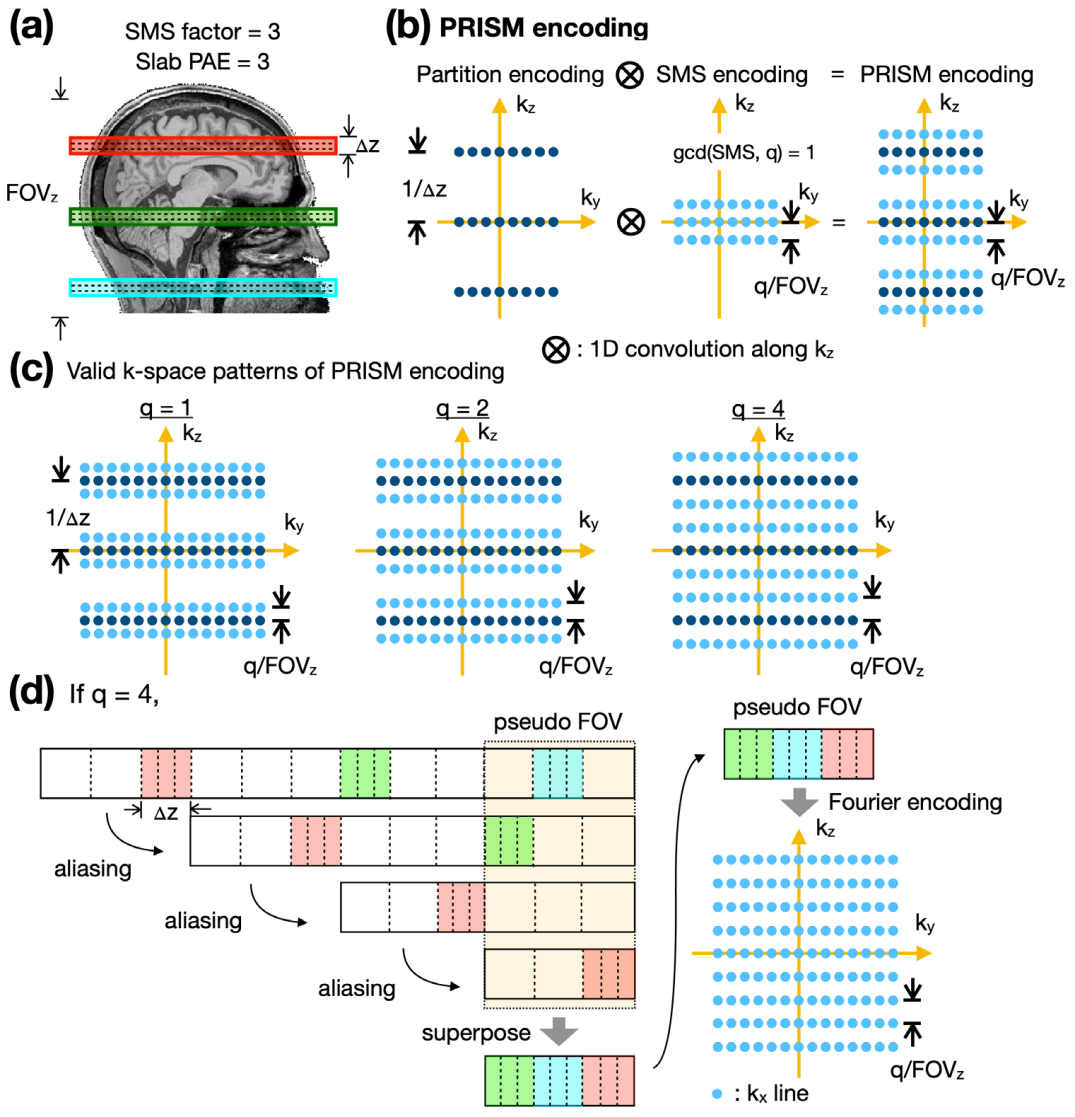
The illustration of PRISM encoding. (a) An example of SMSlab with SMS of 3 and number of PAE = 3. (b) PRISM encoding is achieved through the 1D convolution of partition encoding patterns with SMS encoding patterns along k_z_in k-space. (c) Examples of valid k-space patterns satisfying the condition gcd(SMS, q) = 1. (d) Figurative illustration of simultaneous inter- and intra-slab Fourier encoding. The parameter q is set to 4 in this instance.

To provide a clearer understanding of how PRISM encoding concurrently accomplishes inter-slab and intra-slab Fourier encoding,Figure 1doffers a figurative example for the scenario in which q = 4. We introduce the concept of a “pseudo FOV”, which is highlighted by a dashed box in translucent yellow. The size of pseudo FOV in the z direction is scaled down to FOV_z_/4, accomplished through the k-space sampling pattern illustrated in the right-most panel ofFigure 1c. According to the principles of Digital Fourier Transformation, the reciprocal of the spacing between adjacent k-lines defines the size of the encoded FOV. In accordance with the Nyquist Sampling Theorem, the encoded FOV must be larger than the object to avoid aliasing. When the pseudo FOV is smaller than the object, regions lying outside the pseudo FOV will undergo aliasing, folding back into the confines of the pseudo FOV, as demonstrated in the second row ofFigure 1d. This aliasing process continues iteratively until no regions remain outside the pseudo-FOV. Subsequently, these aliased portions superpose. It is important to note that the aliasing does not occur in a chronological sequence. We describe it step-by-step for illustrative purposes. Due to the presence of gaps and the condition gcd(SMS, q) = 1, the slabs will not overlap (seeSupplementary Materialsfor further details); rather, the three non-contiguous slabs will concatenate into a single, unified volume. In this specific example, the Fourier encoding for SMSlab essentially transforms into a 3D encoding scheme, as highlighted in the bottom right panel ofFigure 1d.

### 2.2 The concept of pseudo slab

InFigure 1, the slab excitation profiles are assumed to be ideal, exhibiting step function at the slab boundary. Unfortunately, an edge transition as sharp as a step function is not possible in reality. Practically, the slab profile will roll over to outside of the predefined slab position as shown in the upper-left panel ofFigure 2. Based on the realistic slab profile, the excitation profile of SMSlab was shown in the upper-mid panel. After the PRISM encoding as shown inFigure 1d, the signal will overlap at the slab boundary as shown in the upper-right panel ofFigure 2.

**Fig. 2.**
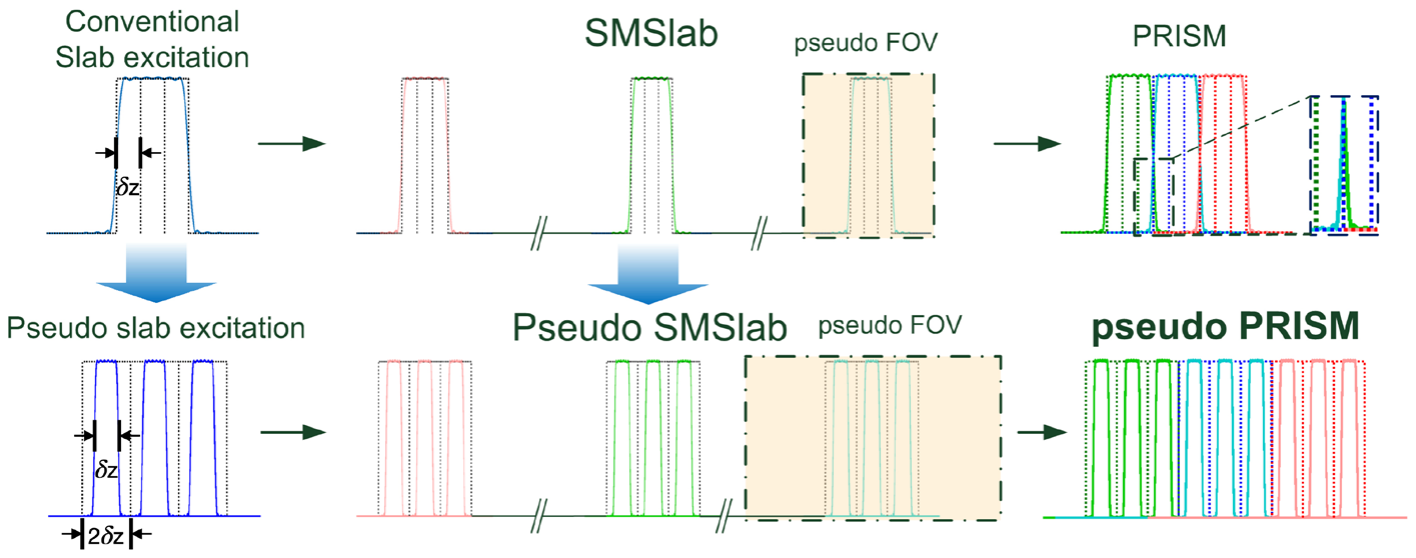
The illustration of pseudo-slab principles. The upper row displays single-slab and SMSlab profiles associated with conventional slab excitation in the left and middle panels, respectively. The δ_z_denotes the slice thickness. The right panel provides an enlarged view of slab overlap at the boundary, following PRISM encoding. A dash-dotted box in translucent yellow highlights the corresponding pseudo FOV. In contrast, the lower row features panels related to pseudo-slab excitation. The excitation profiles for single-slab and SMSlab are shown in the left and middle panels, respectively, while the profile following PRISM encoding is presented in the lower-right panel.

In this study, we propose a concept of a “pseudo slab”, comprising multiple slices separated by a distance equivalent to double the slice thickness, as illustrated in the lower-left panel. The corresponding SMSlab profile is depicted in the lower-middle panel. Due to the existence of intra-slab gaps, the overlap at the slab boundaries following PRISM encoding is effectively minimized, as demonstrated in the lower-right panel. Combining the pseudo slab with the PRISM method, the pseudo PRISM approach is proposed to address the issues of slab-boundary attenuation and high peak RF amplitude.

Additionally, the use of pseudo slabs effectively addresses the challenges associated with conventional slab acquisition. In standard single slab 3D acquisition, the slice thickness is determined by the number of slab PAE. It is imperative for the number of slab PAE to be sufficiently high, typically 8 or more, to mitigate Gibbs truncation artifacts. In the case of a pseudo slab, the partition encoding applied on a pseudo slab is virtually equivalent to implementing phase modulation on each intra-slab slice. Consequently, the Fourier encoding of a pseudo slab is analogous to performing a discrete Fourier transform on each individual slice, which effectively avoids the Gibbs effect. It is important to note that, in the pPRISM method, the slice thickness is determined by the slice excitation profiles rather than the number of partition encoding steps within the slab.

## 3 Materials and Methods

### 3.1 Participants and MR acquisitions

To validate pPRISM method, data were acquired in vivo from two healthy adults with the experimental protocol approved by the Institutional Review Board. Prior to participation, each individual provided written informed consent. MR images were acquired using a Siemens 3T Prisma scanner (Siemens Healthcare, Erlangen, Germany) and a 32-channel head coil at the Biomedical Research Imaging Center (BRIC) at the University of North Carolina at Chapel Hill.

### 3.2 The RF design of single slice

Spatial profiles of magnetization induced by RF pulses were simulated using a Bloch equation simulator in this study (refer tohttp://rsl.stanford.edu/research/software.html). Among various RF pulses, Shinnar–Le Roux (SLR) pulses are commonly utilized due to their effectiveness in achieving the desired slice profile in terms of magnitude. However, SLR pulses do not provide precise control over the phase of the excitation profile across the slice thickness (Pauly et al., 1991). As shown in the upper row inFigure 3, the phase variations of the excitation profile near the two slice boundaries deviate from the desired phase in opposite directions. The peak signal fluctuation within the slice is 37.5%. This deviation can lead to signal cancellation when integrating the through-plane signal during image reconstruction, potentially compromising the SNR. To address this limitation, this study employs the Discrete Inverse Scattering Transform (DIST) technique (Epstein, 2004) to generate excitation pulses with a bandwidth-time (BT) product of 5.4. The excitation profile shown in the lower row indicates that the phase deviation is eliminated, and the within-slice signal fluctuation is lower than 8.3%.

**Fig. 3.**
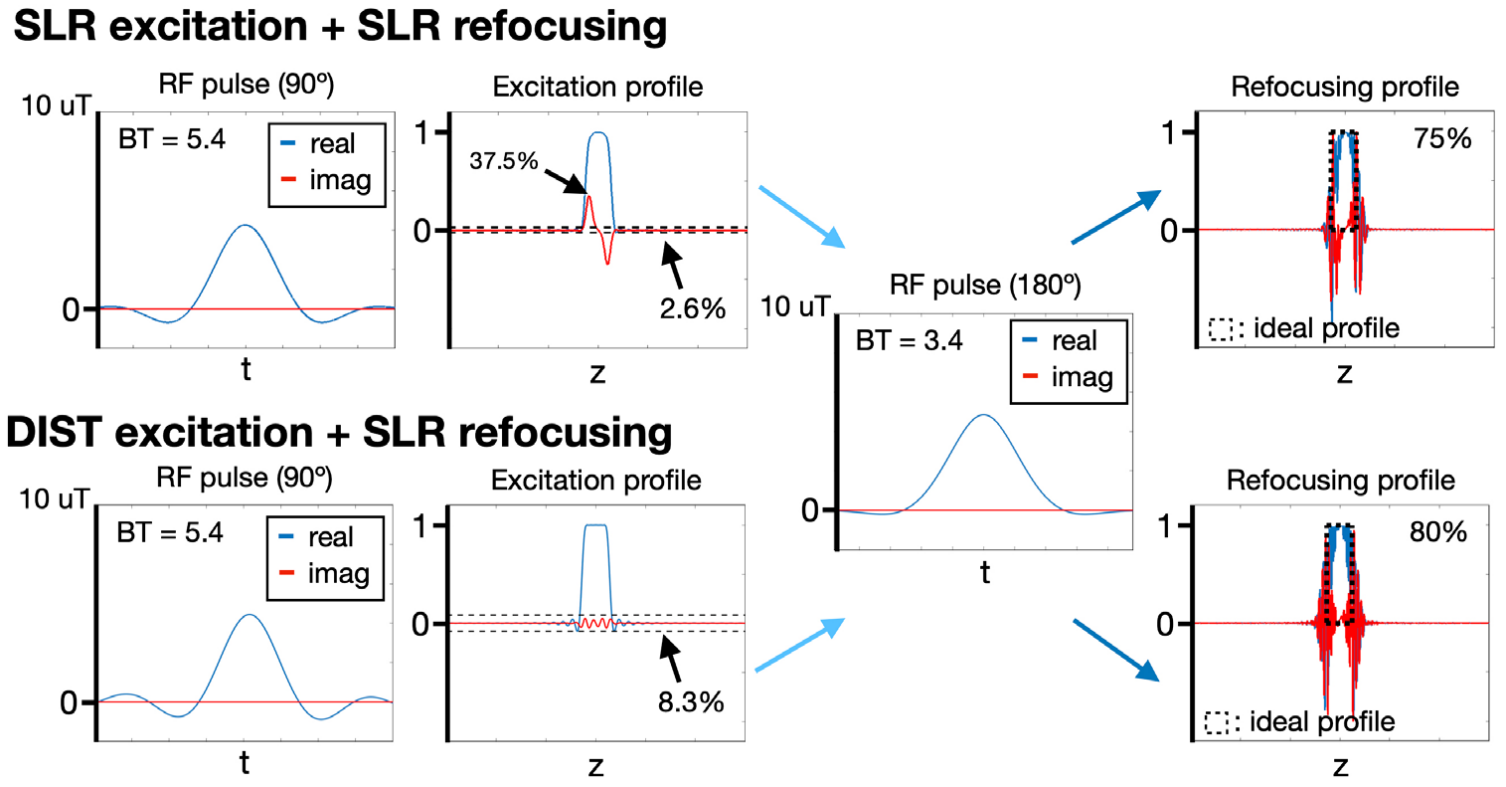
Comparison between SLR and DIST RF excitation pulses. The DIST pulse demonstrates superior phase control within the slice, with a maximum signal fluctuation of less than 8.3%. In contrast, the SLR pulse exhibits a maximum signal fluctuation of 37.5%. The rightmost column shows the resulting spatial profiles after refocusing with a Hamming-windowed SLR pulse. The ideal slice profile is indicated by the dotted black box. The integration of the refocused signal relative to the ideal slice profile is 75% for the SLR pulse and 80% for the DIST pulse.

For refocusing pulse design, DIST pulses were not applicable as their generation becomes impractical when the flip angle is closed to 180º. Therefore, we employed SLR pulses for refocusing. To minimize the peak RF amplitude, a bandwidth-time (BT) product of 3.4 and a Hamming filter were applied. The SLR pulse design was executed using the RF design toolbox (refer tohttp://rsl.stanford.edu/research/software.html) (Pauly et al., 1991). Specifically, the “dzrf” function within the toolbox was utilized, with the pulse type set to “spin echo” and the filter option designated as “Hamming windowed sinc”. Following the RF refocusing, the spatial profiles corresponding to the SLR and DIST pulses are depicted in the upper right and bottom right panels ofFigure 3, respectively. Notably, using the DIST excitation pulse, the integration of the refocused signal within the ideal slice profile (outlined by the black dotted box) achieved 80%, which is 5% higher than using the SLR excitation pulse. Consequently, we employed DIST pulse for the RF design of pseudo slab.

### 3.3 The performance metrics of the RF pulse design

The objective of RF pulse design for a pseudo slab is to optimize the in-slice signal while minimizing the out-of-slice signal following intra-slab decoding. The amplitude of in-slice signal can be quantified by integrating the decoded signal within that specific slice. The out-of-slice signal is assessed by evaluating the signal that originates from a particular slice but disperses to other slices post intra-slab decoding; this is commonly referred to as the Point Spread Function (PSF) caused by slice crosstalk. To calculate the PSF, RF excitation, refocusing, and intra-slab encoding are formulated in a forward matrix, while intra-slab decoding (i.e. inverse Fourier transform) is modeled by an inverse matrix. The spatial resolution of the forward model is 10 times higher than that of the output to avoid the inverse crime (Wirgin, 2004).

The refocused RF profiles are generated using Bloch simulation. Given that a pseudo-slab consists of five pseudo-slices, each being double the thickness of a nominal slice (seeFig. 2), the refocused RF profile is formulated as a 100-by-5 matrix**A**. Each column of matrix**A**represents the RF profile for a specific pseudo-slice. For instance, the RF profile of the first pseudo-slice is represented by the first 20 elements in the first column of**A**, with the remaining elements set to zero. Similarly, the second pseudo-slice is described by elements 21 to 40 in the second column, and so forth. Intra-slab encoding is implemented as a Fourier transform with 5 encoding shots, which yields an encoding matrix**E**of size 5-by-100. These encoded shots are then decoded using the inverse Fourier transform, represented by an inverse matrix**F**^–¹^of size 5-by-5. The PSF is calculated as the product of the matrices**F**^–¹^∙**E**∙**A**. This matrix product forms the resolution matrix**R**, where each column represents the PSF corresponding to a particular source slice. The diagonal elements of R indicate the slice magnitude, while the normalized PSF is obtained by normalizing each column of**R**by its corresponding slice magnitude.

### 3.4 The RF design of pseudo slabs for excitation

**Fig. 4.**
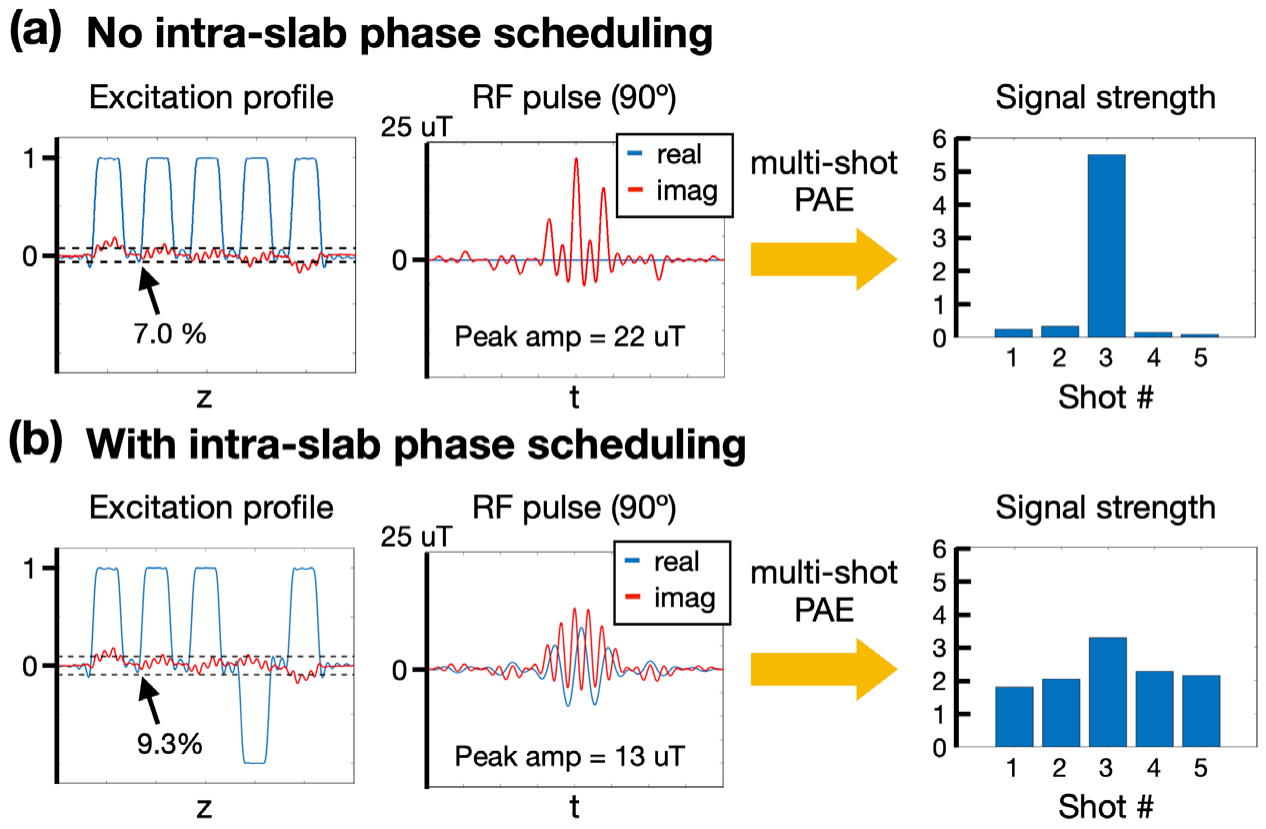
The effect of intra-slab phase scheduling. (a) No intra-slab phase scheduling, (b) With intra-slab phase scheduling. The spatial profiles and RF excitation pulses are shown in the left and middle columns, respectively. Following partition encoding, the signal strength across shots is displayed in the right column.

In the creation of a pseudo slab, phase scheduling is a crucial step that significantly impacts both the peak RF amplitude and the minimum signal intensity across shots. Optimal phase scheduling aims to minimize the peak RF amplitude while maximizing the minimum signal strength across different shots. To achieve this, we conducted an exhaustive search through all possible combinations of modulated phases. We selected phase scheduling sets that fell within the lowest 5% for peak RF amplitude or the highest 5% for minimum signal strength across shots. To expedite this search, the modulated phases were restricted to either 0 or π. If multiple phase scheduling sets met both criteria, we chose the combination that provided the highest minimum signal strength. Conversely, if no combinations met the criteria, we iteratively increased the percentile thresholds from the initial 5% until a suitable phase scheduling combination was identified. Following this optimization process, the optimal phase scheduling set was determined to be (0, 0, 0, π, 0). For the generation of the pseudo slab, we applied modulated phase and frequency shifts to each slice before summation. As illustrated inFigure 4a, the pseudo slab without intra-slab phase scheduling resulted in a high peak RF amplitude and low minimum signal strength across shots. In contrast,Figure 4bdemonstrated that applying intra-slab phase scheduling reduced the peak RF amplitude by 41% and increased the minimum signal strength by 18-fold.

### 3.5 The RF design of pseudo slabs for refocusing

In the design of pseudo slabs for refocusing, it might seem intuitive to apply the same phase scheduling to the refocusing pulse as is used for the excitation pulse. However, the Bloch simulations suggest that this approach leads to signal oscillation in the gaps between intra-slab slices, as illustrated by the gray arrows inFigure 5a. This oscillation is attributed to constructive interference when two adjacent slices share the same polarity. Empirically, the oscillated signal in the slice gaps caused artificial signal enhancement as highlighted by the red arrows inFigure 5a. While incorporating the Bloch-simulated PSF into the reconstruction process (seeSection 3.9for details) can mitigate the striping artifacts, as shown in the right-most panel inFigure 5a, it does not completely eliminate them. Note that the images inFigure 5were reconstructed using an ideal zero phase rather than phase self-navigation to avoid confounding effects from imperfect phase estimation.

**Fig. 5.**
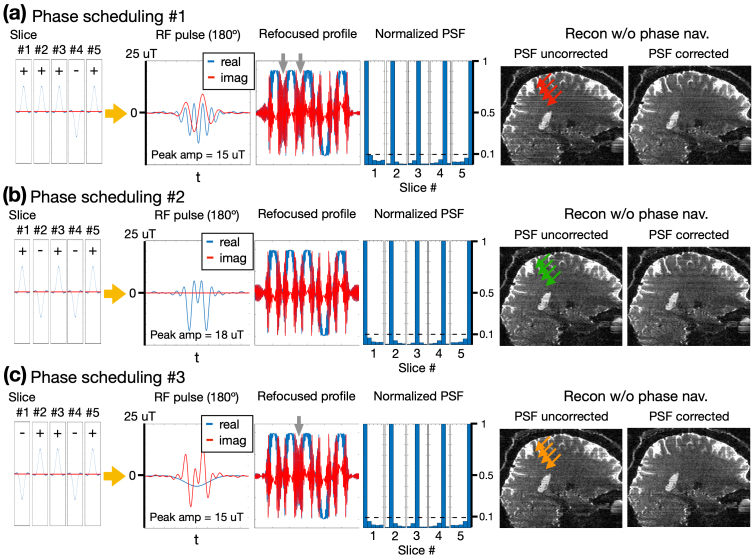
Examination of three types of phase scheduling for refocusing pulses. The three different phase scheduling combinations are corresponding to different pulse polarities: (a) + + + - +, (b) + - + - +, (c) - + + - + . The second, third, and fourth columns from the left display the RF pulses, spatial profiles, and point-spread functions (PSF) respectively. The gray arrows indicate the oscillated signal in the slice gaps. The 5^th^and 6^th^columns display the pPRISM images without and with PSF correction respectively. The red and orange arrows in the 5^th^column indicate the artificial signal enhancement by the oscillated signal in the slice gaps while the green arrows denote the absence of artifacts. All the pPRISM images were reconstructed using an ideal zero phase rather than phase self-navigation to eliminate confounding effects from imperfect phase estimation during self-navigation.

To address this issue, we leveraged the fact that signal refocusing can be accomplished by either polarity of refocusing pulse, allowing for the polarity design to minimize between-slice signal oscillation with minimal increase in peak RF amplitude.Figure 5bshows that by assigning opposite polarities to any pair of adjacent slices, the refocused profile exhibits the least signal oscillation within the gaps. The in vivo test on the right ofFigure 5balso demonstrated the reduction of artificial stripes, as highlighted by the green arrows. This adjustment, however, led to a 20% increase in peak RF amplitude. A compromise solution involved modifying the pulse polarities to (-, +, +, -, +) as shown inFigure 5c, which effectively reduced the number of slice gaps exhibiting signal oscillation from two to one, while maintaining the same peak RF amplitude. The resulting point-spread functions (PSFs) in the right panels were kept below 10%, as indicated by the dashed black lines. After PSF correction, the striping artifacts can be largely mitigated. Therefore, the refocusing pulse shown inFigure 5cwas used for the rest of the study.

**Fig. 6.**
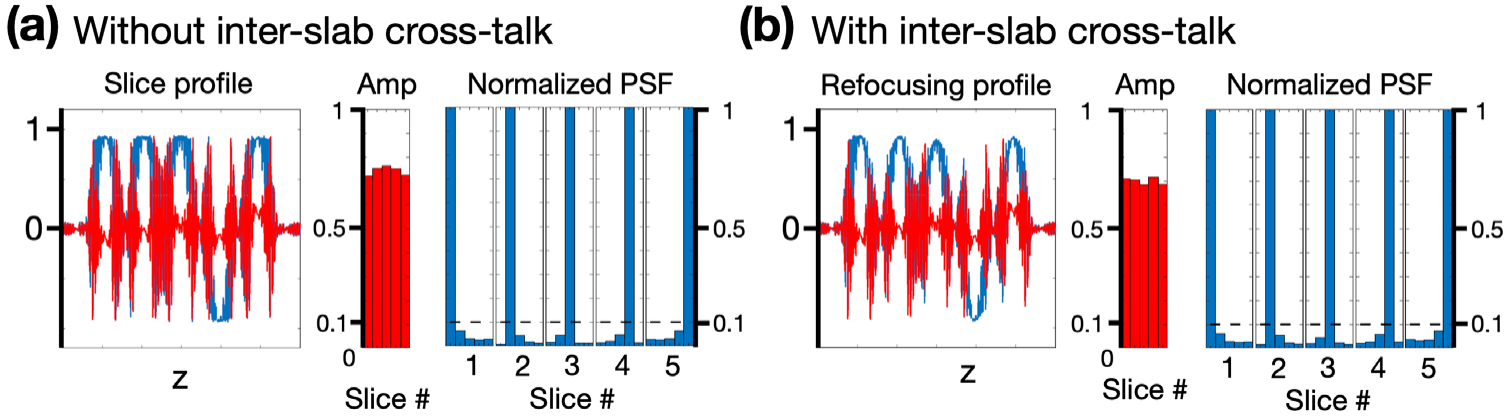
The Bloch-simulated slice profile, signal amplitude, and normalized PSF (a) without and (b) with inter-slab cross-talk. The TR is set to 3.5 s.

In addition to the intra-slab cross-talk demonstrated inFigure 5, RF excitation and refocusing from interleaved groups can also introduce cross-talk, referred to as inter-slab cross-talk. To evaluate the impact of inter-slab cross-talk, Bloch simulations were conducted to compare the slice profile, signal amplitude, and PSF under conditions with and without interleaved RF excitation and refocusing. Assuming a TR of 3.5 s, the Bloch-simulated results without inter-slab cross-talk are shown inFigure 6a, where RF excitation and refocusing from the other interleaved group were omitted to eliminate any inter-slab cross-talk. In contrast,Figure 6bshows the Bloch-simulated results with RF excitation and refocusing from both interleaved groups. Quantitatively, the averaged signal amplitude inFigure 6bis reduced by 5.7% compared to that inFigure 6a. For the PSFs, the profiles with and without interleaved RF excitation and refocusing across all intra-slab slices remain highly similar. The results indicated that inter-slab cross-talk resulted in a slight signal reduction but had a negligible impact on the PSFs.

### 3.6 The RF design of pPRISM pulses

During the generation of simultaneous multi-pseudo slabs, we will implement an additional phase scheduling strategy as proposed byWong (2012). The inter-slab phase scheduling will be applied to each pseudo slab in conjunction with intra-slab phase scheduling to further reduce the peak RF amplitude. For SMS factor of 2, the modulated phases for the two pseudo slabs are 0 and π.

### 3.7 Acquisitions of pPRISM

Based on the fully-sampled PRISM encoding, the design of an accelerated sampling pattern is straightforward, as illustrated inFigure 7a. Assuming an SMS factor of 2 and a PAE number of 5, the fully-sampled pattern is represented by light-gray circles. Utilizing the blipped-Controlled Aliasing-in-Parallel-Imaging (blipped-CAIPI) technique, the corresponding accelerated patterns for each imaging shot are represented by solid dots of distinct colors.

**Fig. 7.**
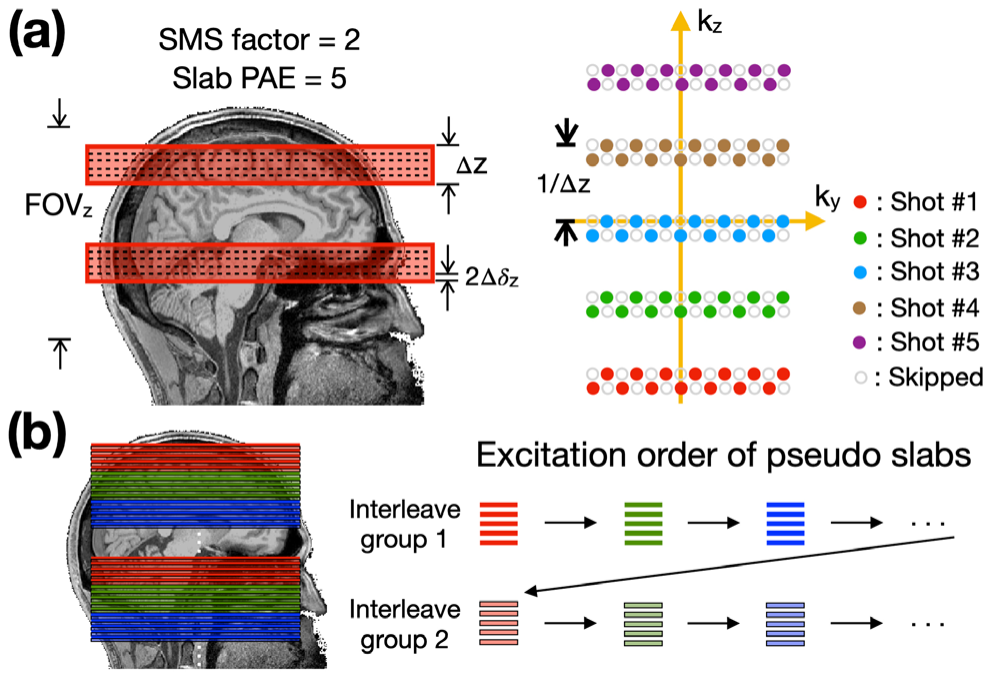
Acquisition strategies for pPRISM. (a) Simultaneous multi-slab with SMS = 2 and a number of PAE = 5. The left panel displays the excitation volumes, while the right panel depicts the sampling trajectories. Δz denotes the thickness of pseudo slab and δ_z_denotes the slice thickness. Circles in the right panel indicate the skipped k_x_lines, while solid dots indicate the sampling trajectories. Different colors correspond to different shots. (b) Excitation orders of pseudo slabs for volumetric acquisition. Darker and lighter colors correspond to the first and second interleaving groups, respectively.

Figure 7billustrates the slice-interleaving strategy for pPRISM acquisition for the whole-brain coverage. The first interleaving group acquires pseudo slab sequentially across the entire FOV, starting from one extreme to the other (either top-to-bottom or bottom-to-top), as represented by darker colors in the figure. Subsequently, the second interleaving group acquires the slices residing in the interstitial gaps within pseudo slabs following a sequential pattern, as represented by lighter colors in the figure.

### 3.8 The image reconstruction of pseudo slab

The image reconstruction procedure generally includes two steps. The first step is slab reconstruction and the second step is slice reconstruction, as shown inFigure 8. In the first step, individual pseudo slabs will be unaliased from the simultaneously-excited pseudo slabs using SENSitivity Encoding (SENSE)-based reconstruction. The coil sensitivity profile was estimated from the central k-space lines of a reference image using ESPIRiT (Uecker et al., 2014). Specifically, the command is “ecalib -r 24 -m 1 -d 7 -c 0.5” in Berkeley Advanced Reconstruction Toolbox (BART) toolbox (https://mrirecon.github.io/bart/index.html). Let**y**, N_shot_and N_ch_denote the acquired data, the number of shots and number of coil receiving channels.

**Fig. 8.**
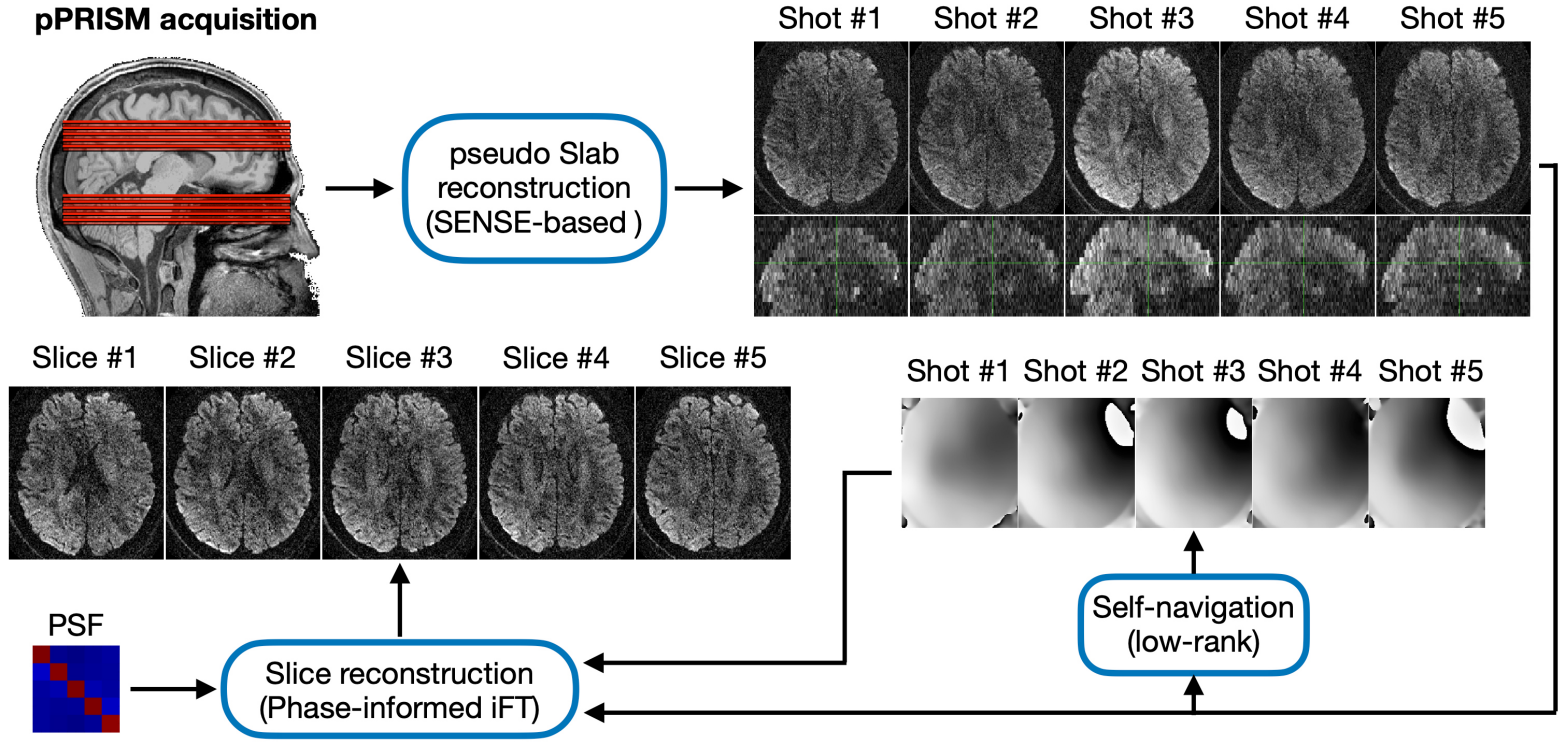
Image reconstruction procedure for pPRISM imaging. Initially, simultaneously-acquired pseudo slabs are unaliased into individual pseudo slabs corresponding to different partition encoding (PAE) shots using a SENSE-based approach. Subsequently, the phase images for each shot are estimated through a low-rank approach. Finally, the intra-slab slices are reconstructed using these estimated phase images, the inverse Fourier transform (iFT) and PSF. The PSF is derived from Bloch simulation.

$$y={\left[y_{1}^{\text{T}},y_{2}^{\text{T}},\ldots y_{{\text{N}}_{\text{shot}}}^{\text{T}}\right]}^{\text{T}}$$ (1)

$$y_{i}={\left[{\left(y_{i}^{1}\right)}^{\text{T}},{\left(y_{i}^{2}\right)}^{\text{T}},\ldots {\left(y_{i}^{{\text{N}}_{\text{ch}}}\right)}^{\text{T}}\right]}^{\text{T}}$$ (2)

where*i*= 1, 2,… N_shot_and [∙]^T^denotes the transpose operation. For each shot,$y_{i}^{j}=E\cdot C^{j}\cdot x_{i}$where*j*= 1, 2,… N_ch_and**x***_i_*denotes the*i*-th shot image of a pseudo slab that we seek to reconstruct. The encoding matrixEincorporates the phase encoding and blipped-CAPI encoding.

$$E=[E^{1},\;\;E^{2},\ldots E^{{\text{N}}_{\text{sms}}}]$$ (3)

$${\text{x}}_{i}={\left[x_{i}^{1},\;x_{i}^{2},\ldots \;\;x_{i}^{{\text{N}}_{\text{sms}}}\right]}^{T}$$ (4)

where N_sms_denotes the SMS factor. The blipped-CAIPI encoding is dependent on the central position of the slab along z direction. With the data**y**collected from all the coils, we can reconstruct the collapsed images of each pseudo slab for every acquisition shot by solvingEq. (5)using conjugate gradient method “pcg” in MATLAB.

$${\hat{x}}_{i}=\arg\underset{{\text{x}}_{i}}{\min\;}\;{\left\|{\text{y}}_{i}-\;\;\text{E}\cdot \text{C}\cdot {\text{x}}_{i}\right\|}_{2}^{2},$$ (5)

The upper row inFigure 8presents an example of reconstructed pseudo slabs**x***_i_*. Consistent with the bar charts inFigure 4b, the 3rd shot exhibited a slightly stronger signal compared to the other shots. All shots demonstrated adequate SNR for the subsequent self-navigation step.

### 3.9 The self-navigation and reconstruction of slice images

The second step is to reconstruct the intra-slab slices for each pseudo slab. The phase images of each shot will be estimated first before the slices can be reconstructed. Assuming a slowly varying in-plane phase, the phase information can be accurately approximated using low k-space information. Therefore, the images were 4x down-sampled in x-y plane, which, in turn, provided a much shorter reconstruction time. We also employed structured low-rank matrix completion approach for multi-shot phase estimation based on the annihilation relations between shots (Haldar, 2014;Mani et al., 2020). The formulation for the reconstruction of intra-slab slices is as follows:

$${\hat{\text{s}}}_{ds}^{k}=\arg\underset{{\text{s}}_{ds}^{k}}{\min}{\left\|\;{\text{x}}_{ds}^{k}-\text{P}\cdot {\text{s}}_{ds}^{k}\right\|}_{2}^{2}\;\;+\;\;\lambda_{1}{\left\|\;\text{H}\left({\text{s}}_{ds}^{k}\right)\right\|}_{*}+\lambda_{2}{\left\|\;{\text{s}}_{ds}^{k}\right\|}_{2}^{2},$$ (6)

$${\text{x}}_{ds}^{k}={\left[{\left({\text{x}}_{ds,1}^{k}\right)}^{\text{T}},{\left({\text{x}}_{ds,2}^{k}\right)}^{\text{T}},\ldots {\left({\text{x}}_{ds,N_{shot}}^{k}\right)}^{\text{T}}\right]}^{\text{T}},$$ (7)

$${\text{s}}_{ds}^{k}={\left[{\left({\text{s}}_{ds,1}^{k}\right)}^{\text{T}},{\left({\text{s}}_{ds,2}^{k}\right)}^{\text{T}},\ldots {\left({\text{s}}_{ds,N_{shot}}^{k}\right)}^{\text{T}}\right]}^{\text{T}},$$ (8)

where*k*= 1, 2,… N_slab_. The subscript “ds” denotes the down-sampled version. N_slab_denotes the total number of pseudo slabs.**P**denotes the forward matrix that incorporates partition encoding, phase scheduling and PSF. The PSF, derived from Bloch simulations (seeSection 3.3for details), accounts for steady-state conditions, TR, and crosstalk between interleaved slice groups. In these simulations, a T1 value of 1200 ms was used. Due to the proximity of slices within a pseudo-slab, slice crosstalk is inevitable. Incorporating the PSF into the image reconstruction process is essential to effectively mitigate the striping artifacts resulting from these crosstalk effects.H(·)is an operation that generates block-Hankel matrix of the shots. Specifically, we employed the Low-Rank Modeling of Local-Space Neighborhoods (LORAKS) to impose a phase constraint, given that the image phase varies slowly (Haldar, 2014).${\left\|\;\cdot \;\right\|}_{*}$denotes the nuclear norm.$\lambda_{1}$and$\lambda_{2}$are the regularization parameters.

$$\lambda_{1}\;\;\;=\;\;0.01\;\;\cdot \;\;{\left\|\;{\text{x}}_{ds}^{k}\right\|}_{2}^{2}/{\left\|\;\text{H}\text{(}{\text{s}}_{ds,ini}^{k})\;\right\|}_{*},$$ (9)

$$\lambda_{2}=0.1\;\;\cdot \;\;{\left\|\;{\text{x}}_{ds}^{k}\right\|}_{2}^{2}/{\left\|\;{\text{s}}_{ds,ini}^{k}\right\|}_{2}^{2},$$ (10)

where${\text{s}}_{ds,ini}^{k}$is the initial solution in (6) without the regularization terms. The scalar values of 0.01 and 0.1 inEq. (9)andEq. (10), respectively, were obtained through in vivo testing. To improve the quality of phase estimation, the estimated intra-slab slices${\hat{\text{s}}}_{ds}^{k}$were averaged within a pseudo slab and up-sampled, assuming the motion-induced phase varies slowly within a pseudo slab. Ultimately, the phase derived from the mean image was incorporated into the reconstruction of individual slice images.

$${\hat{\text{s}}}^{k}=\arg\underset{{\text{s}}^{k}}{\min}\;{\left\|\;{\hat{\text{x}}}^{k}-\text{P}\cdot {\hat{\Phi }}^{k}\cdot {\text{s}}^{k}\right\|}_{2}^{2}+\lambda \;{\left\|\;{\text{s}}^{k}\right\|}_{2}^{2},$$ (11)

where λ=0.1/N_shot_and${\hat{\Phi }}^{k}$denotes the estimated phase derived fromEq. (6). The**P**denotes the matrix that models partition encoding, intra-slab phase scheduling and PSF. The conjugate gradient method “pcg” in MATLAB was used as the solver forEq. (6)and(11).

The reconstructed images can be further denoised using NOise Reduction with DIstribution Corrected principal component analysis (NORDIC PCA) method (Vizioli et al., 2021). This procedure was implemented using the “NORDIC” function in the NORDIC toolbox, and the g-factor maps were incorporated. The g-factor maps were calculated using an analytic approach (Robson et al., 2008). Notably, the images fed into the function were of complex value.

### 3.10 The imaging protocols

In this study, two datasets with submillimeter isotropic resolutions of 0.86 mm and 0.76 mm were obtained. The SMS factor was 2 and number of PAE shots was 5 for both datasets. The phase encoding was along the anterior-posterior (AP) direction, while the PAE direction was aligned superior-inferior (SI). The partial Fourier factor is 6/8. A monopolar diffusion acquisition was performed. DIST RF pulses with a duration of 8 ms and BT product of 5.4 were used for 90° excitations and an SLR RF pulse with Hamming filter, BT product of 3.4, and pulse duration of 9.6 ms was used for 180° refocusing. Please see earlier sections for technical details.

For the dataset with 0.86-mm resolution, the in-plane image dimensions were set to 224 x 256. The TE is 90 ms and in-plane acceleration rate is 2. For comparison, the gSlider images with the same in-plane image protocol were also acquired. We selected the gSlider technique for its use of multishot, navigator-free methods, which provide a relatively short acquisition time per volume compared to other 3D multishot techniques. The gSlider sequence was received from Massachusetts General Hospital (MGH) under Siemens customer-to-Producer (C2P) agreement. At a TR of 3.5 s, the total numbers of slabs for the pPRISM and gSlider sequences were 32 and 30 respectively, translating to total slice counts of 160 and 150. When the TR was reduced to 2.4 s, the slice counts for pPRISM and gSlider were adjusted to 140 and 130, respectively.

For the 0.76-mm resolution dataset, the in-plane image dimensions were 256 x 256, and the in-plane acceleration was increased to 3. The TEs were set at 80 ms and 89 ms for b-values of 1000 and 2500, respectively. The total number of slabs was 36, yielding 180 slices. The TR was 3 s. To assess the impact of spatial resolution on imaging quality, a comparison dataset with a coarser resolution of 1.5 mm isotropic was acquired using a single-shot SMS sequence from the Center for Magnetic Resonance Research (CMRR) at the University of Minnesota. The in-plane dimensions for this lower resolution set were 128 x 140, with no in-plane acceleration, an SMS factor of 4, and a total of 92 slices.

### 3.11 Diffusion analysis

In this study, the three dMRI datasets with isotropic resolutions of 1.5 mm, 0.86 mm, and 0.76 mm were processed by MRtrix3 (Tournier et al., 2019). The processing steps may include gradient checking, motion and eddy current correction, and streamline tractography. For ODF estimation, we employed the “Dhollander” algorithm for unsupervised estimation of response functions for white matter (WM), gray matter (GM), and cerebral spinal fluid (CSF). Additionally, the “Tournier” algorithm was used for single-fiber voxel selection and response function estimation. Streamline tractography was performed using spherical deconvolution with the MRtrix3 command “trkgen”. A 4th-order Runge-Kutta integration (option “-rk4”) was used, with a minimum streamline length of 20 mm (option “-minlength”). To ensure uniform seed distribution, the “-seed_grid_per_voxel” option was applied, using a grid of 3 seeds per voxel for the submillimeter datasets and 6 seeds per voxel for the 1.5 mm dataset, enabling more comprehensive tract coverage. The fiber tracking data was then refined using the command “tcksift”, ensuring that streamline densities corresponded accurately with the fiber-orientation-distribution (FOD) lobe integrals. Finally, the streamline tractography was visualized in “mrview”, with the colors of the streamlines determined by their mean orientations at endpoints.

## 4 Results

### 4.1 The image quality of pPRISM and the comparison with gSlider

The reconstructed pPRISM images are presented inFigure 9, with a spatial resolution of 0.86 mm isotropic and 5 PRISM encoding shots. The TR per shot is 2.4 s, and the effective TR is 12 s. To evaluate the impact of phase self-navigation, we compared the b0 images without and with phase self-navigation, as shown in the first and second columns from the left. When phase self-navigation was disabled, the phase was set to zero.

**Fig. 9.**
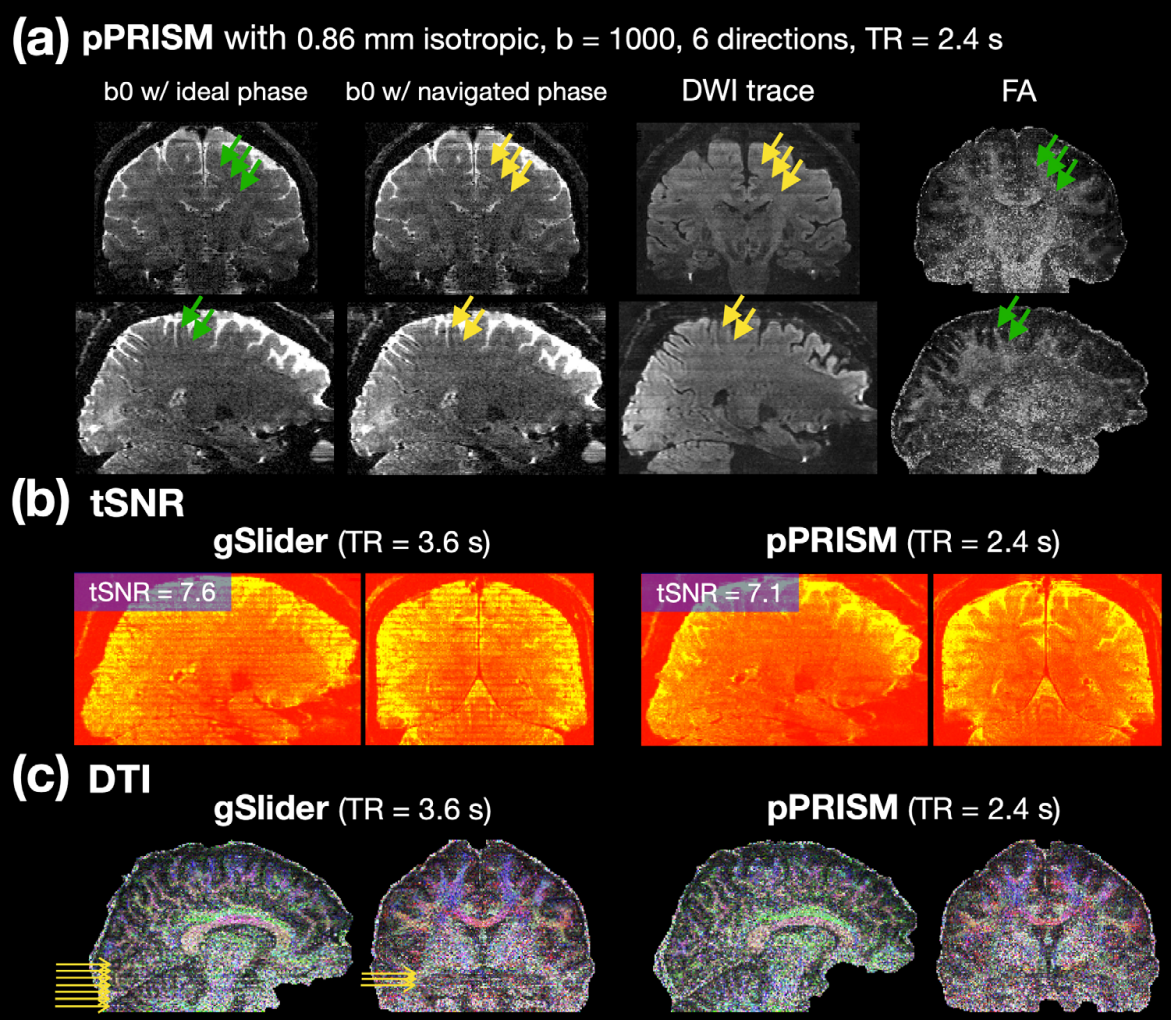
The image quality of pPRISM and the comparison with gSlider. (a) pPRISM images with a spatial resolution of 0.86 mm and a TR of 2.4 s (effective TR of 12 s). The first and second columns show the b0 images with ideal zero phase and self-navigated phase, respectively. The third column presents the trace image, averaged across six diffusion directions, and the right-most column displays the FA maps. Yellow arrows highlight observable striping artifacts, while green arrows point to the same locations where the striping artifacts are less noticeable. (b) tSNR maps for gSlider and pPRISM, calculated from a time series of b0 images. The TRs for gSlider and pPRISM are 3.6 s and 2.4 s, respectively. The brain-averaged tSNRs for gSlider and pPRISM are 7.6 and 7.1, respectively. (c) Diffusion tensor imaging (DTI) results for gSlider and pPRISM. Six diffusion directions were acquired, with two repetitions for gSlider and three repetitions for pPRISM. Yellow arrows indicate the presence of striping artifacts.

Slight slab boundary artifacts were observed when phase self-navigation was employed, as indicated by the yellow arrows in the 2nd column ofFigure 9a. These artifacts were minimized when the ideal zero phase was applied, as shown by the green arrows in the 1st column ofFigure 9a. These findings suggest that imperfect phase self-navigation can lead to minor slab boundary artifacts. Nevertheless, similar slab boundary artifacts were also observed in the diffusion-weighted images (DWIs), as demonstrated in the trace image in the third column. The trace images were averaged across six diffusion directions. Since the diffusion tensor image (DTI) is computed based on the signal attenuation in the DWIs relative to the b0 image, the slab boundary artifacts caused by imperfect phase navigation do not negatively affect the subsequent diffusion analysis, as indicated by the green arrows in the fourth column ofFigure 9a.

We also aimed to investigate SNR efficiency and compare it with gSlider images. Recognizing that noise distribution can vary spatially across the image, we assessed the noise standard deviation by measuring temporal variation at each voxel, rather than measuring noise outside of the brain. To this end, we acquired a time series of b0 images using both gSlider and pPRISM, matched for scan time. Specifically, 24 gSlider b0 images with a TR of 3.6 s and 36 pPRISM b0 images with a TR of 2.4 s were acquired. The SNR maps were calculated using temporal SNR (tSNR). As shown inFigure 9b, the tSNR of gSlider is slightly higher than that of pPRISM by 7%. However, since pPRISM can acquire 50% more volumes than gSlider within the same time frame, the SNR can be improved by 22.3% using the pPRISM method. Therefore, the SNR efficiency of pPRISM is 14.3% higher than that of gSlider imaging. Furthermore, gSlider demonstrated substantial variation in SNR between slab-boundary and non-slab-boundary slices, resulting in striping artifacts in DTI, as indicated by the yellow arrows inFigure 9c. In contrast, pPRISM provided a more homogeneous SNR distribution across slices, resulting in significantly fewer striping artifacts in DTI, as shown in the right panel ofFigure 9c.

**Fig. 10.**
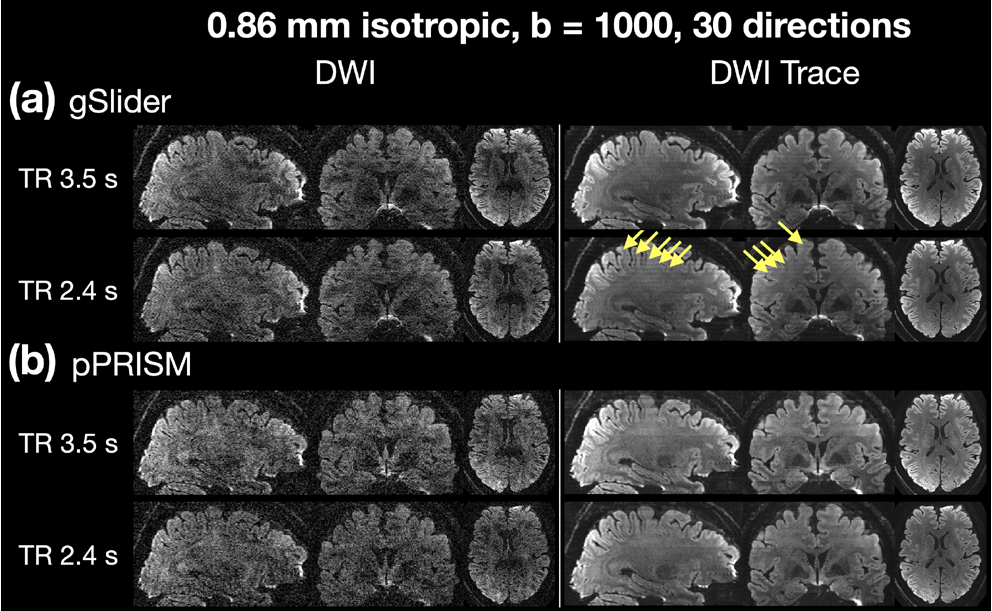
Comparison of image quality between (a) gSlider and (b) pPRISM methods. The spatial resolution is 0.86 mm isotropic and b-value is 1000 s/mm^2^. The SMS factor is 2 and the number of shots is 5. The number of diffusion directions is 30. The yellow arrows indicate the slab boundary artifacts. As the TR is reduced from 3.5 s to 2.4 s, the signal attenuation at slab boundaries become pronounced in gSlider images, while pPRISM images show minimal change.

Figure 10compares the effects of shortened TR on both pPRISM and gSlider. The DWIs are shown on the left, while the trace DWIs are shown on the right. Both sets of images have a spatial resolution of 0.86 mm isotropic and were acquired with a b-value of 1000 s/mm² using 30 diffusion directions. In the gSlider images, slab-boundary attenuation is not evident when the TR is 3.5 s but becomes pronounced at a TR of 2.4 s, as shown inFigure 10a. In contrast, the pPRISM images inFigure 10bexhibit no significant slab-boundary attenuation regardless of the TR value, demonstrating the superior performance of pPRISM in maintaining consistent image quality across different TR settings.

Although slab-boundary artifacts were not evident in the pPRISM images, striping artifacts were noticeable around the top and middle regions of the brain in the DWI trace images shown inFigure 10b. These artifacts are similar to those observed inFigure 9a, indicating that the primary source is unlikely to be related to RF excitation or refocusing. Instead, these artifacts are most likely due to residual errors in phase navigation.

**Fig. 11.**
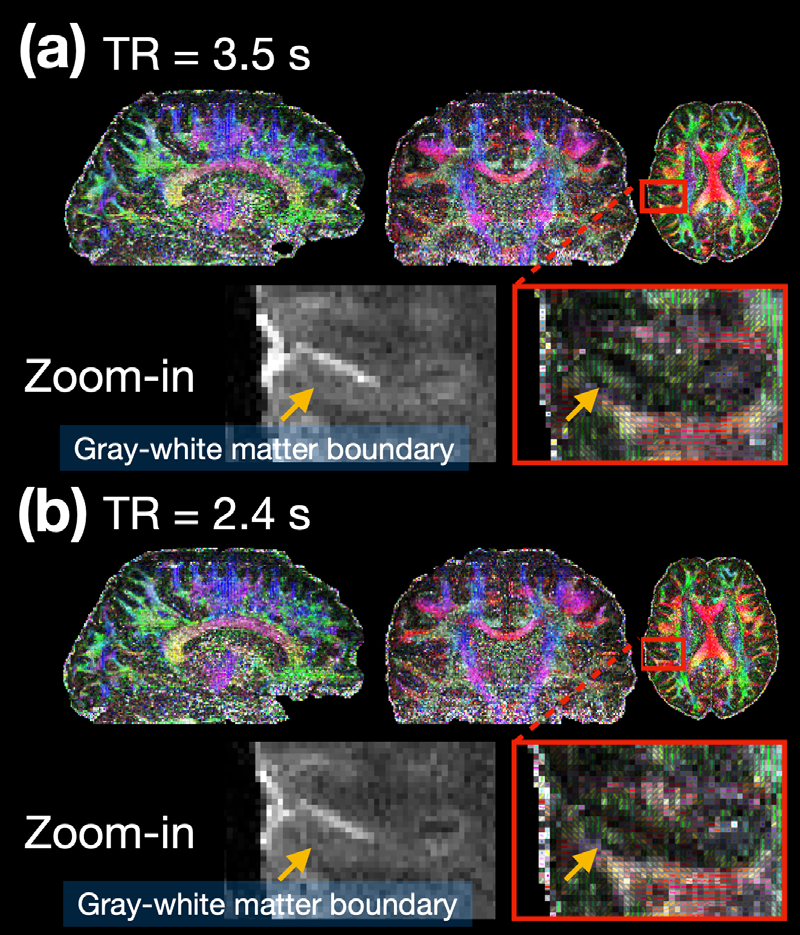
Effect of shortened acquisition time on the pPRISM method. (a) TR of 3.5 s and (b) TR of 2.4 s. The spatial resolution is 0.86 mm isotropic with a b-value of 1000 s/mm^2^. The SMS factor is 2 and the number of shots is 5. The upper row shows the color-coded FA maps, while the lower row provides zoomed-in views of the highlighted regions, along with b0 images for better identification of gray-white matter boundaries.

AlthoughFigure 10demonstrates minimal slab-boundary attenuation irrespective of the TR duration for pPRISM method, a shorter TR reduces signal amplitude due to decreased T1 recovery time, as seen in the lower row ofFigure 10b. It is therefore important to assess the extent to which this signal reduction affects subsequent diffusion analysis.Figure 11shows color-coded FA maps for pPRISM with (a) TR of 3.5 s and (b) TR of 2.4 s. For enhanced visualization, the cortical regions highlighted by the red boxes are enlarged in the lower rows, along with enlarged b0 images for reference. In both figures, dark bands at the gray-white matter boundaries, indicated by light brown arrows, are observed. However, the results with TR of 3.5s appear more spatially stable compared to those with TR of 2.4 s.

### 4.2 Comparison of sensitivity to B0 field inhomogeneity

For the gSlider method, the peak RF amplitude exceeds the system threshold if constant slice-selection gradient is used. To address this, the Variable-rate selective excitation (VERSE) method is employed to reduce the peak RF amplitude. The “VERSEd” RF pulses and gradients were synthesized using the minimum time VERSE toolbox (https://github.com/mriphysics/verse-mb). Nevertheless, the VERSE method renders the sequence sensitive to B_0_field inhomogeneity, whose effects can be modeled using a Bloch equation simulator. As shown inFigure 12a, the slice amplitude at the slab boundary in gSlider is notably attenuated. Furthermore, off-resonance conditions have led to an elevation in the normalized PSF, exceeding 10% in four of the five intra-slab slices. In contrast, the spatial profiles and slice amplitudes of pPRISM shown inFigure 12bexhibit virtually no alterations in comparison to the results inFigure 5c. The normalized PSFs are consistently maintained well below the 10% threshold.

**Fig. 12.**
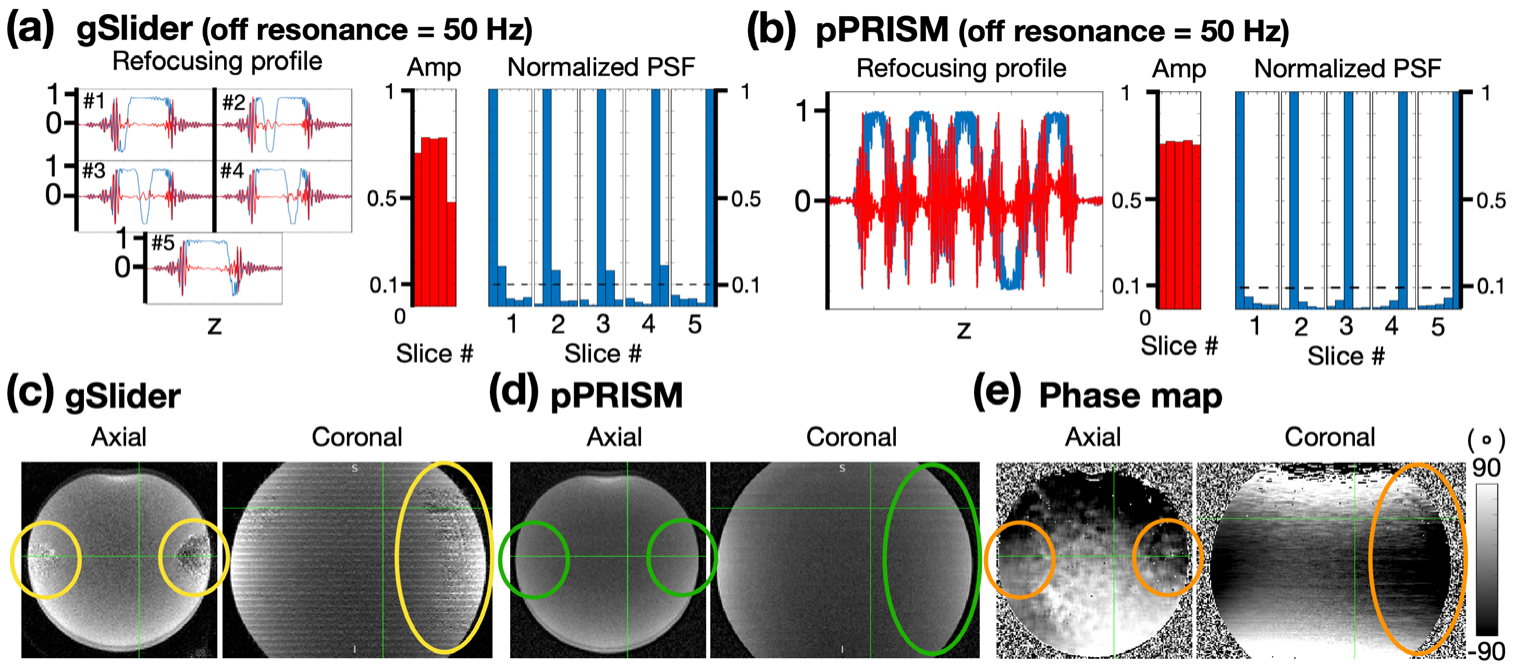
Simulated and empirical effects of off-resonance on gSlider and pPRISM. Panels (a) and (b) show the results of Bloch simulations for gSlider and pPRISM, respectively, with an off-resonance frequency of 50 Hz applied. The spatial profiles, slice amplitudes, and point spread functions (PSFs) are displayed from left to right, with dashed lines marking the 10% thresholds. Panels (c) and (d) present the empirical phantom images for gSlider and pPRISM, while (e) displays the corresponding phase map. The yellow, green, and orange circles highlight the regions corresponding to the artifacts in (c).

In addition to Bloch simulation, we also performed the empirical comparison between gSlider and pPRISM. To avoid potential head movement and the complexity of brain geometry, we conducted a phantom experiment instead of an in vivo test. The phantom used was spherical in shape. InFigure 12c, the gSlider image exhibits artifacts, as highlighted by the yellow circles. In contrast, the pPRISM image inFigure 12dshows no such artifacts, as indicated by the green circles. To investigate the source of those artifacts in gSlider images, we obtained the phase map from fully-sampled reference image of pPRISM. The phase map shown inFigure 12edemonstrated inhomogeneous phase within the regions marked by the orange circles, suggesting that the artifacts observed in the gSlider image may be attributed to field inhomogeneity in those areas.

### 4.3 Diffusion analyses of pPRISM with spatial resolution of 0.76 mm isotropic

We further evaluate the performance of pPRISM at a spatial resolution of 0.76 mm and b-values up to 2500 s/mm^2^. This protocol involved acquiring two-shell datasets with b-values of 1000 and 2500, each comprising 64 diffusion directions. Total number of diffusion directions is 128. The overall acquisition time is approximately 33 minutes. The NORDIC method was employed for denoising. To assess the performance, the maps of colored FA, Orientation Distribution Function (ODF), and tractography were computed. The diffusion-weighted images with b-values of 1000 and 2500 are shown inFigure 13a. The colored-FA maps were generated using DWIs from both b-values, incorporating all 128 diffusion directions. Similar to the analysis inFigure 11, we evaluated the ability to detect fine structures at this resolution by examining the dark bands at gray-white matter boundaries. As illustrated in the enlarged views within the red and green boxes inFigure 13b, the dark bands at gray-white matter boundaries can be clearly observed in both axial and coronal views, as indicated by the brown arrows.

**Fig. 13.**
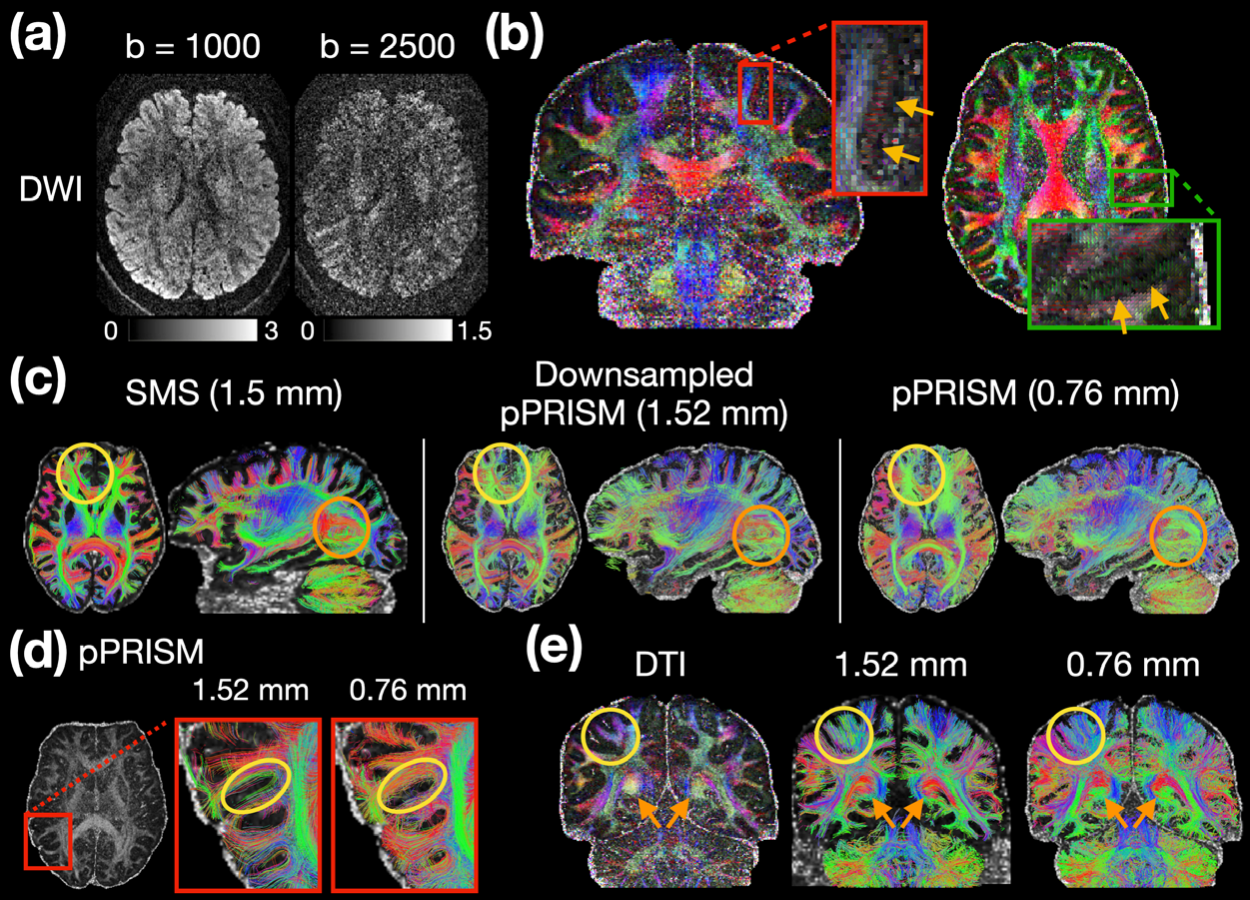
Diffusion results from the 0.76 mm isotropic data. (a) Diffusion-weighted images (DWIs) at b-values of 1000 and 2500 s/mm^2^. (b) Color-coded FA maps, calculated from two-shell DWIs with b-values of 1000 and 2500 s/mm^2^, each with 64 diffusion directions. Zoomed-in tensor results within the gray matter are shown in coronal and axial views. Brown arrows point to the dark bands at the gray-white matter boundaries. (c) Tractography maps at 1.5-mm SMS, down-sampled 1.52-mm pPRISM, and 0.76-mm pPRISM resolutions. (d) Enlarged view comparing U-fiber visibility in the 1.52-mm and 0.76-mm pPRISM maps. (e) DTI and tractography maps at 1.52-mm and 0.76-mm resolution. Yellow circles and orange arrows highlight key differences in fiber orientation and anatomical plausibility.

Figure 13ccompares the tractography maps at resolutions of 1.5 mm, 1.52 mm, and 0.76 mm, displayed from left to right. The colors of the streamlines are determined by the mean orientation of their endpoints. The 1.52-mm resolution images were generated by down-sampling the 0.76-mm dataset by a factor of two in order to examine the partial volume effect. Overall, all tractography maps exhibit similar patterns. However, the 0.76-mm pPRISM imaging revealed more fiber tracks compared to both the 1.52-mm down-sampled pPRISM and the 1.5-mm SMS images. The higher resolution also improved the plausibility of the streamline tractography. For example, the orange circles inFigure 13chighlight the posterior arcuate fasciculus (AF), which connects Broca’s area and Wernicke’s area in the brain. As the AF primarily runs in the anterior-posterior direction, it is expected to be color-coded green in tractography, which is the color shown in the 0.76-mm pPRISM images. However, in both the 1.5-mm SMS and 1.52-mm down-sampled pPRISM images, the posterior AF is color-coded red, which is less plausible. Another example is the area highlighted by the yellow circles, which is part of the forceps minor. The forceps minor crosses the midline via the genu of the corpus callosum, connecting the left and right frontal lobes. The encoded color is expected to be green. The 0.76-mm pPRISM images display the most anatomically plausible green coloring, whereas the 1.5-mm SMS and 1.52-mm down-sampled maps exhibit less plausible red coloring. This suggests that crossing fibers between the forceps minor and the genu of the corpus callosum are not well-resolved in the lower-resolution datasets. Additionally, the similarity between the 1.5-mm SMS and 1.52-mm down-sampled pPRISM tractography maps supports that the differences observed between 0.76-mm pPRISM and 1.5-mm SMS imaging are largely due to partial volume effects.

U-fiber visibility is commonly regarded as a hallmark of submillimeter dMRI performance. InFigure 13d, the 0.76-mm pPRISM tractography reveals more U-fibers than the down-sampled 1.52-mm tractography. Moreover, in the yellow-circled region, the cortex is laterally oriented in the axial view. This orientation makes it more likely for the U-fibers beneath the cortex to be color-coded red, as seen in the 0.76-mm tractography. In contrast, the 1.52-mm tractography shows some U-fibers encoded as green, which is less anatomically plausible.

Additional discrepancies between the 1.52-mm and 0.76-mm tractography maps were also observed. InFigure 13e, the DTI results in the left column show that the fibers within the yellow circles run in the superior-inferior direction, suggesting that these regions are part of the corticospinal tract, which is typically expected to appear in a blueish color. This is reflected in the 0.76-mm tractography map, but not in the 1.52-mm tractography map. Furthermore, the orange arrows in the DTI map highlight the forceps major, shown in a yellowish color. Similar to the forceps minor, the forceps major crosses the midline via the splenium of the corpus callosum, connecting the left and right occipital lobes. The forceps major is expected to be color-coded green, as seen in the 0.76-mm tractography map. However, this is not observed in the 1.52-mm tractography map.

## 5 Discussion

The Fourier encoding of simultaneously-excited slabs was deemed unattainable using gradient coding alone (Dai et al., 2019). This study proposed an encoding strategy, PRISM, capable of achieving both inter-slab and intra-slab encoding exclusively using the gradient. Additionally, we proposed the concept of a pseudo slab to minimize inter-slab signal leakage and Gibbs truncation effect. The employment of the pseudo slab enables the application of phase scheduling to intra-slab slices. The intra-slab phase scheduling reduces the peak RF amplitude and enhances the minimum signal strength across shots, thereby eliminating the need for time-varying gradients and phase navigators. By employing the pPRISM technique, which integrates pseudo slab and PRISM methods, we accomplished dMRI with a 0.76-mm isotropic resolution, an effective TR of 15 s (TR of 3s per shot), and b-values of 2500 s/mm^2^. The artifacts at the slab boundaries are minimal, and structural details, such as the dark band at grey-white matter boundary, are discernible.

The principles of PRISM encoding were initially identified through exhaustive search. Subsequent to this discovery, the encoding rules depicted inFigure 1bwere formalized. These rules were later validated mathematically, as detailed in theSupplementary Materials. Conceptually, PRISM encoding represents a subsampling pattern in 3D sampling, wherein the FOV spans the entirety of the SMSlab. Naturally, subsampling within k-space results in aliasing in the image space. The PRISM methodology provides specific k-space sampling guidelines that exploit the gaps present between the SMSlabs, ensuring that any FOV aliasing does not result in overlapping slabs. Additionally, PRISM encoding demonstrates that multiple patterns are capable of achieving orthogonal encoding for SMSlab, as illustrated inFigure 1c. In cartesian k-space sampling, the pattern corresponding to q = 1 was selected owing to its minimal blip size in the z direction. For non-cartesian k-space sampling methods, such as radial or spiral trajectories, patterns exhibiting a more uniform distribution across k-space might be preferable.

The concept of pseudo-slab concept allows for the implementation of phase scheduling within a pseudo slab, thereby eliminating the need for a phase navigator. In conventional 3D slab imaging, shots associated with high spatial frequency, particularly those with larger k_z_values, exhibit low signal intensity. This necessitates the use of a phase navigator to estimate the image phase for each shot, consequently leading to increased SAR and prolonged acquisition times per shot. In contrast, optimal phase scheduling increases the minimum signal intensity across shots as demonstrated inFigures 4band5, while maintaining a relatively low peak RF amplitude. However, it’s noteworthy that our study confined the optimization of phase scheduling to situations where modulated phases were either 0 orπ. While this approach facilitated a quicker search, it is possible that a superior phase scheduling could further enhance signal intensity across shots and reduce peak RF amplitude. Exploration of this possibility is deferred to future studies.

While pPRISM demonstrated decent performance, some other methodologies may refine the image quality further. In this study, SENSitivity Encoding (SENSE)-based reconstruction was used (Pruessmann et al., 1999). Generally, adopting Generalized Autocalibrating Partial Parallel Acquisition (GRAPPA)-based reconstructions (Griswold et al., 2002) might incrementally enhance SNR (Robson et al., 2008). Additionally, introducing virtual coil concept into the reconstruction could reduce the g-factor penalty (Blaimer et al., 2009). The corresponding shifted sampling scheme could also be employed in the pPRISM sequence to further improve the image quality (Blaimer et al., 2009). Moreover, the blip-up/down acquisition (BUDA) method is complementary to the pPRISM method and could alleviate geometric distortion issues (Liao et al., 2021). Future work will integrate these techniques to optimize the image quality.

To render diffusion MRI with submillimeter isotropic resolution practicable for clinical research, it is imperative to ensure acquisition times remain reasonably short. In recent years, the integration of 3D multi-slab multi-shot EPI acquisition with multiplexed sensitivity encoded (MUSE) reconstruction (Chen et al., 2013) facilitated the achievement of a 0.85-mm isotropic resolution with an acquisition time of 36 s per volume and a maximal b-value of 800 s/mm^2^(Chang et al., 2015). Subsequently, the gSlider SMS approach substantially improved the performance, achieving isotropic resolution of 0.76 mm, acquisition time per volume of 17.5 s, and maximal b-value of 2500 s/mm^2^(Wang et al., 2021). Our proposed pPRISM method not only matches the aforementioned resolution and maximal b-value but also reduces the acquisition time per volume to 15 s. Additionally, the pPRISM approach exhibited the resilience against slab boundary artifacts and field inhomogeneity. Our pPRISM technique represents a promising advancement in ultrahigh-resolution diffusion MRI, offering both efficiency and robustness.

### 5.1 Limitations

During the self-navigation procedure, the intra-slab slices across all shots represent unknown parameters. Specifically, the total number of unknowns is proportional to the square of the shot count, while the equation count is linearly dependent on the number of shots. This disparity renders the self-navigation as an ill-posed problem. To address this underdetermined situation, this investigation utilized low-rank regularization techniques. While we utilized the linear interdependence between shots, certain factors remained unexploited: 1) the consistent motion-induced phase across intra-slab slices, and 2) the consistent magnitude of slices throughout the shots. Without harnessing this knowledge to constrain the solutions in the self-navigation procedure, the phase estimation may be imprecise, and residual errors across shots may persist. For example, slight striping artifacts at the top edge and middle of brain are observable in the trace DWI images inFigure 10b. In those axial slices at the top edge of the brain, the brain tissues occupy only a relatively small portion of the slice image, making phase estimation in those slices challenging. Nevertheless, these striping artifacts have minimal impact on subsequent diffusion analyses, as demonstrated by the results presented inFigures 9and13.

The number of unknowns in the self-navigation procedure could potentially be reduced from a quadratic relationship with the shot count to a linear one by integrating the aforementioned prior knowledge into the optimization framework. Specifically, the unknown count would become 2 × N_shot_, including N_shot_of slab phase images and N_shot_of slice magnitude images. However, this transformation would reframe the problem as a nonlinear optimization one. For gSlider imaging, the initial phase estimates have shown reliable accuracy (Haldar et al., 2020), facilitating rapid convergence. For pPRISM imaging, an imprecise initial phase estimate might lead to extended convergence times in a nonlinear optimization setting. Currently, using low-rank regularization for phase estimation across the entire volume takes less than 10 minutes on our institutional servers. Introducing nonlinear optimization could significantly extend this reconstruction time. While reducing the number of unknowns may improve reconstruction accuracy, its feasibility is contingent upon substantial reductions in computational time.

Given that the proposed pPRISM approach functions as a 2D method rather than a true 3D approach, the resolution along the slice direction is determined by the RF excitation and refocusing profiles, rather than by spatial encoding. Specifically, the achievable slice thickness is dependent on the maximum gradient strength and the pulse duration. On our Siemens Prisma scanner, the minimum slice thickness is limited to 0.556 mm when using a refocusing pulse duration of 9.6 ms. Achieving higher resolution in the slice direction would require extending the pulse duration, which would increase the echo time (TE) and consequently reduce the SNR. Furthermore, extending the pulse duration reduces the RF bandwidth if the same time-bandwidth product is maintained, making thinner slices more susceptible to field inhomogeneities (Hargreaves et al., 2011;Hopper et al., 2006;Lu et al., 2009). While increasing the time-bandwidth product can alleviate this susceptibility by broadening the RF bandwidth, it also elevates the peak RF amplitude and gradient strength. These parameters are constrained by the RF amplifier’s power limits and the gradient system’s specifications. Therefore, users should carefully balance between minimizing slice thickness and preserving adequate SNR and image quality when targeting higher resolutions. Additionally, reversed slice-select gradient acquisition was recently proposed to correct Additionally, a reversed slice-select gradient acquisition has recently been proposed to correct geometric distortions in the slice direction (Blazejewska et al., 2022).

Although pPRISM has significantly reduced the intensity differences between shots by more than 15-fold, as demonstrated inFigure 4, the intensity of the zero-encoding shot (e.g., the 3rd shot inFig. 4b) remains approximately 50% higher than the others. In principle, these intensity variations should not affect the final reconstructed image as long as they are incorporated into the optimization process. However, differences in SNR across shots could influence the accuracy of phase self-navigation. The low SNR in some shots could introduce errors in phase estimation, potentially affecting the resulting image quality. Potential solution is to apply the global scaling across shots so that the median voxel intensity remains the same, such as by incorporating a scaling factor inEq. (7)(Haldar et al., 2020). We will explore this approach in the future.

The use of the pPRISM method in the submillimeter resolution regime inherently increases sensitivity to head motion. While inter-volume head motion can be effectively corrected retrospectively (Tournier et al., 2019), addressing intra-volume motion remains a more complex challenge. A promising solution for this issue is the motion-corrected gSlider (MC-gSlider) technique (Wang et al., 2018), developed for the gSlider framework. In MC-gSlider, motion parameters are iteratively estimated by registering RF-encoded diffusion volumes to the corresponding b0 reference image with the same RF encoding. These parameters are then incorporated into the gSlider image reconstruction, resulting in a motion-aware reconstruction process. The effectiveness of this approach is attributed to the intra-slab encoding scheme, which allows for high-SNR images to be reconstructed directly from individual slab-encoding shots. This implies that the approach is not applicable for 3D multi-slab or volumetric imaging. Given the pPRISM method also produces slab-encoded images with decent SNR, this motion-correction approach has strong potential to be adapted for pPRISM. We plan to investigate this adaptation in future research.

The computation speed is a concern in our current implementation. For the 0.86-mm images with 30 diffusion directions, the pseudo slab reconstruction and phase self-navigation required approximately 3.5 and 4 hours, respectively. The entire image reconstruction process was carried out using unoptimized MATLAB code on shared Linux-based workstations available campus-wide (https://help.rc.unc.edu/getting-started-on-longleaf#system-information). We employed MATLAB’s “parfor” function to facilitate parallel computing with 12 CPUs. For the 0.76-mm images with 64 diffusion directions, the pseudo slab reconstruction and self-navigation took approximately 10 and 11.5 hours, respectively. The computation speed could be significantly enhanced if the algorithm were implemented in C++, although optimizing the code is beyond the scope of this study.

## 6 Conclusion

By utilizing the proposed pPRISM method, this research achieved submillimeter-resolution dMRI with minimized slab boundary artifacts and improved SNR efficiency while also demonstrating robustness to field inhomogeneity. We anticipate that the pPRISM technique will significantly advance research in technically challenging regions, such as the cortical layers within the entorhinal cortex, thereby paving the way for clinical microstructural research.

## Supplementary Material

Supplementary Material

## Data Availability

All data supporting the findings of this study are made publicly available athttps://openneuro.org/datasets/ds005737. This ensures that other researchers can replicate, verify, and build upon the work presented. The computational code will also be made available to interested parties. Please note that the code is provided in its raw form and has not been optimized, cleaned, or extensively commented. While this may impact ease of use or adaptation, we believe it remains a valuable resource for those interested in understanding or extending our analytical methods.

## References

[b1] Assaf , Y. ( 2019 ). Imaging laminar structures in the gray matter with diffusion MRI . NeuroImage , 197 , 677 – 688 . 10.1016/j.neuroimage.2017.12.096 29309898

[b2] Blaimer , M. , Gutberlet , M. , Kellman , P. , Breuer , F. A. , Köstler , H. , & Griswold , M. A. ( 2009 ). Virtual coil concept for improved parallel MRI employing conjugate symmetric signals . Magnetic Resonance in Medicine , 61 ( 1 ), 93 – 102 . 10.1002/mrm.21652 19097211

[b3] Blazejewska , A. I. , Witzel , T. , Andersson , J. L. R. , Wald , L. L. , & Polimeni , J. R. ( 2022 ). Slice-direction geometric distortion evaluation and correction with reversed slice-select gradient acquisitions . NeuroImage , 264 , 119701 . 10.1016/j.neuroimage.2022.119701 36283542 PMC9910288

[b4] Block , K. T. , Uecker , M. , & Frahm , J. ( 2008 ). Suppression of MRI truncation artifacts using total variation constrained data extrapolation . International Journal of Biomedical Imaging , 2008 , 184123 . 10.1155/2008/184123 18784847 PMC2531202

[b5] Bruce , I. P. , Chang , H.-C. , Petty , C. , Chen , N.-K. , & Song , A. W. ( 2017 ). 3D-MB-MUSE: A robust 3D multi-slab, multi-band and multi-shot reconstruction approach for ultrahigh resolution diffusion MRI . NeuroImage , 159 , 46 – 56 . 10.1016/j.neuroimage.2017.07.035 28732674 PMC5676310

[b6] Chang , H.-C. , Sundman , M. , Petit , L. , Guhaniyogi , S. , Chu , M.-L. , Petty , C. , Song , A. W. , & Chen , N. ( 2015 ). Human brain diffusion tensor imaging at submillimeter isotropic resolution on a 3 Tesla clinical MRI scanner . NeuroImage , 118 , 667 – 675 . 10.1016/j.neuroimage.2015.06.016 26072250 PMC4554968

[b7] Chen , N. , Guidon , A. , Chang , H.-C. , & Song , A. W. ( 2013 ). A robust multi-shot scan strategy for high-resolution diffusion weighted MRI enabled by multiplexed sensitivity-encoding (MUSE) . NeuroImage , 72 , 41 – 47 . 10.1016/j.neuroimage.2013.01.038 23370063 PMC3602151

[b8] Dai , E. , Wu , Y. , Wu , W. , Guo , R. , Liu , S. , Miller , K. L. , Zhang , Z. , & Guo , H. ( 2019 ). A 3D k‐space Fourier encoding and reconstruction framework for simultaneous multi‐slab acquisition . Magnetic Resonance in Medicine , 82 ( 3 ), 1012 – 1024 . 10.1002/mrm.27793 31045283 PMC6831486

[b9] Engström , M. , & Skare , S. ( 2013 ). Diffusion-weighted 3D multislab echo planar imaging for high signal-to-noise ratio efficiency and isotropic image resolution: Diffusion-weighted 3DMS-EPI . Magnetic Resonance in Medicine , 70 ( 6 ), 1507 – 1514 . 10.1002/mrm.24594 23359357

[b10] Epstein , C. L. ( 2004 ). Minimum energy pulse synthesis via the inverse scattering transform . Journal of Magnetic Resonance , 167 ( 2 ), 185 – 210 . 10.1016/j.jmr.2003.12.014 15040975

[b11] Frank , L. R. , Jung , Y. , Inati , S. , Tyszka , J. M. , & Wong , E. C. ( 2010 ). High efficiency, low distortion 3D diffusion tensor imaging with variable density spiral fast spin echoes (3D DW VDS RARE) . NeuroImage , 49 ( 2 ), 1510 – 1523 . 10.1016/j.neuroimage.2009.09.010 19778618 PMC2791091

[b12] Frost , R. , Miller , K. L. , Tijssen , R. H. N. , Porter , D. A. , & Jezzard , P. ( 2014 ). 3D Multi-slab diffusion-weighted readout-segmented EPI with real-time cardiac-reordered k-space acquisition: 3D Multi-Slab rs-EPI . Magnetic Resonance in Medicine , 72 ( 6 ), 1565 – 1579 . 10.1002/mrm.25062 24347093

[b13] Glover , G. H. , & Chang , C. ( 2012 ). Hadamard-encoded sub-slice fMRI for reduced signal dropout . Magnetic Resonance Imaging , 30 ( 1 ), 1 – 8 . 10.1016/j.mri.2011.07.019 21937181

[b14] Griswold , M. A. , Jakob , P. M. , Heidemann , R. M. , Nittka , M. , Jellus , V. , Wang , J. , Kiefer , B. , & Haase , A. ( 2002 ). Generalized autocalibrating partially parallel acquisitions (GRAPPA) . Magnetic Resonance in Medicine , 47 ( 6 ), 1202 – 1210 . 10.1002/mrm.10171 12111967

[b15] Gulban , O. F. , De Martino , F. , Vu , A. T. , Yacoub , E. , Uğurbil , K. , & Lenglet , C. ( 2018 ). Cortical fibers orientation mapping using in-vivo whole brain 7 T diffusion MRI . NeuroImage , 178 , 104 – 118 . 10.1016/j.neuroimage.2018.05.010 29753105 PMC6118131

[b16] Haldar , J. P. ( 2014 ). Low-rank modeling of local *k* -space neighborhoods (LORAKS) for constrained MRI . IEEE Transactions on Medical Imaging , 33 ( 3 ), 668 – 681 . 10.1109/TMI.2013.2293974 24595341 PMC4122573

[b17] Haldar , J. P. , Liu , Y. , Liao , C. , Fan , Q. , & Setsompop , K. ( 2020 ). Fast submillimeter diffusion MRI using gSlider-SMS and SNR-enhancing joint reconstruction . Magnetic Resonance in Medicine , 84 ( 2 ), 762 – 776 . 10.1002/mrm.28172 31919908 PMC7968733

[b18] Hargreaves , B. A. , Cunningham , C. H. , Nishimura , D. G. , & Conolly , S. M. ( 2004 ). Variable-rate selective excitation for rapid MRI sequences . Magnetic Resonance in Medicine , 52 ( 3 ), 590 – 597 . 10.1002/mrm.20168 15334579

[b19] Hargreaves , B. A. , Worters , P. W. , Pauly , K. B. , Pauly , J. M. , Koch , K. M. , & Gold , G. E. ( 2011 ). Metal-induced artifacts in MRI . American Journal of Roentgenology , 197 ( 3 ), 547 – 555 . 10.2214/AJR.11.7364 21862795 PMC5562503

[b20] Hopper , T. A. J. , Vasilić , B. , Pope , J. M. , Jones , C. E. , Epstein , C. L. , Song , H. K. , & Wehrli , F. W. ( 2006 ). Experimental and computational analyses of the effects of slice distortion from a metallic sphere in an MRI phantom . Magnetic Resonance Imaging , 24 ( 8 ), 1077 – 1085 . 10.1016/j.mri.2006.04.019 16997078

[b21] Liao , C. , Bilgic , B. , Tian , Q. , Stockmann , J. P. , Cao , X. , Fan , Q. , Iyer , S. S. , Wang , F. , Ngamsombat , C. , Lo , W. , Manhard , M. K. , Huang , S. Y. , Wald , L. L. , & Setsompop , K. ( 2021 ). Distortion‐free, high‐isotropic‐resolution diffusion MRI with gSlider BUDA‐EPI and multicoil dynamic B _0_ shimming . Magnetic Resonance in Medicine , 86 ( 2 ), 791 – 803 . 10.1002/mrm.28748 33748985 PMC8121182

[b22] Lu , W. , Pauly , K. B. , Gold , G. E. , Pauly , J. M. , & Hargreaves , B. A. ( 2009 ). SEMAC: Slice encoding for metal artifact correction in MRI . Magnetic Resonance in Medicine , 62 ( 1 ), 66 – 76 . 10.1002/mrm.21967 19267347 PMC2837371

[b23] Mani , M. , Jacob , M. , McKinnon , G. , Yang , B. , Rutt , B. , Kerr , A. , & Magnotta , V. ( 2020 ). SMS MUSSELS: A navigator‐free reconstruction for simultaneous multi‐slice‐accelerated multi‐shot diffusion weighted imaging . Magnetic Resonance in Medicine , 83 ( 1 ), 154 – 169 . 10.1002/mrm.27924 31403223 PMC7895319

[b24] McNab , J. A. , Polimeni , J. R. , Wang , R. , Augustinack , J. C. , Fujimoto , K. , Stevens , A. , Janssens , T. , Farivar , R. , Folkerth , R. D. , Vanduffel , W. , & Wald , L. L. ( 2013 ). Surface based analysis of diffusion orientation for identifying architectonic domains in the in vivo human cortex . NeuroImage , 69 , 87 – 100 . 10.1016/j.neuroimage.2012.11.065 23247190 PMC3557597

[b25] Miller , K. L. , Stagg , C. J. , Douaud , G. , Jbabdi , S. , Smith , S. M. , Behrens , T. E. J. , Jenkinson , M. , Chance , S. A. , Esiri , M. M. , Voets , N. L. , Jenkinson , N. , Aziz , T. Z. , Turner , M. R. , Johansen-Berg , H. , & McNab , J. A. ( 2011 ). Diffusion imaging of whole, post-mortem human brains on a clinical MRI scanner . NeuroImage , 57 ( 1 ), 167 – 181 . 10.1016/j.neuroimage.2011.03.070 21473920 PMC3115068

[b26] Pauly , J. , Le Roux , P., Nishimura , D. , & Macovski , A. ( 1991 ). Parameter relations for the Shinnar-Le Roux selective excitation pulse design algorithm (NMR imaging) . IEEE Transactions on Medical Imaging , 10 ( 1 ), 53 – 65 . 10.1109/42.75611 18222800

[b27] Pruessmann , K. P. , Weiger , M. , Scheidegger , M. B. , & Boesiger , P. ( 1999 ). SENSE: Sensitivity encoding for fast MRI . Magnetic Resonance in Medicine , 42 ( 5 ), 952 – 962 . 10.1002/(SICI)1522-2594(199911)42:5<952::AID-MRM16>3.0.CO;2-S 10542355

[b28] Robson , P. M. , Grant , A. K. , Madhuranthakam , A. J. , Lattanzi , R. , Sodickson , D. K. , & McKenzie , C. A. ( 2008 ). Comprehensive quantification of signal-to-noise ratio and *g* -factor for image-based and *k* -space-based parallel imaging reconstructions . Magnetic Resonance in Medicine , 60 ( 4 ), 895 – 907 . 10.1002/mrm.21728 18816810 PMC2838249

[b29] Setsompop , K. , Cohen-Adad , J. , Gagoski , B. A. , Raij , T. , Yendiki , A. , Keil , B. , Wedeen , V. J. , & Wald , L. L. ( 2012 ). Improving diffusion MRI using simultaneous multi-slice echo planar imaging . Neuroimage , 63 ( 1 ), 569 – 580 . 10.1016/j.neuroimage.2012.06.033 22732564 PMC3429710

[b30] Setsompop , K. , Fan , Q. , Stockmann , J. , Bilgic , B. , Huang , S. , Cauley , S. F. , Nummenmaa , A. , Wang , F. , Rathi , Y. , Witzel , T. , & Wald , L. L. ( 2018 ). High-resolution in vivo diffusion imaging of the human brain with generalized slice dithered enhanced resolution: Simultaneous multislice (gSlider-SMS) . Magnetic Resonance in Medicine , 79 ( 1 ), 141 – 151 . 10.1002/mrm.26653 28261904 PMC5585027

[b31] Tournier , J.-D. , Smith , R. , Raffelt , D. , Tabbara , R. , Dhollander , T. , Pietsch , M. , Christiaens , D. , Jeurissen , B. , Yeh , C.-H. , & Connelly , A. ( 2019 ). MRtrix3: A fast, flexible and open software framework for medical image processing and visualisation . NeuroImage , 202 , 116137 . 10.1016/j.neuroimage.2019.116137 31473352

[b32] Uecker , M. , Lai , P. , Murphy , M. J. , Virtue , P. , Elad , M. , Pauly , J. M. , Vasanawala , S. S. , & Lustig , M. ( 2014 ). ESPIRiT—An eigenvalue approach to autocalibrating parallel MRI: Where SENSE meets GRAPPA . Magnetic Resonance in Medicine , 71 ( 3 ), 990 – 1001 . 10.1002/mrm.24751 23649942 PMC4142121

[b33] Van , A. T. , Aksoy , M. , Holdsworth , S. J. , Kopeinigg , D. , Vos , S. B. , & Bammer , R. ( 2015 ). Slab profile encoding (PEN) for minimizing slab boundary artifact in three-dimensional diffusion-weighted multislab acquisition: Slab profile encoding . Magnetic Resonance in Medicine , 73 ( 2 ), 605 – 613 . 10.1002/mrm.25169 24691843 PMC4182345

[b34] Vizioli , L. , Moeller , S. , Dowdle , L. , Akçakaya , M. , De Martino , F. , Yacoub , E. , & Uğurbil , K. ( 2021 ). Lowering the thermal noise barrier in functional brain mapping with magnetic resonance imaging . Nature Communications , 12 ( 1 ), Article 1. 10.1038/s41467-021-25431-8 PMC8405721 34462435

[b35] Wang , F. , Bilgic , B. , Dong , Z. , Manhard , M. K. , Ohringer , N. , Zhao , B. , Haskell , M. , Cauley , S. F. , Fan , Q. , Witzel , T. , Adalsteinsson , E. , Wald , L. L. , & Setsompop , K. ( 2018 ). Motion‐robust sub‐millimeter isotropic diffusion imaging through motion corrected generalized slice dithered enhanced resolution (MC‐gSlider) acquisition . Magnetic Resonance in Medicine , 80 ( 5 ), 1891 – 1906 . 10.1002/mrm.27196 29607548 PMC6107445

[b36] Wang , F. , Dong , Z. , Tian , Q. , Liao , C. , Fan , Q. , Hoge , W. S. , Keil , B. , Polimeni , J. R. , Wald , L. L. , Huang , S. Y. , & Setsompop , K. ( 2021 ). In vivo human whole-brain Connectom diffusion MRI dataset at 760 µm isotropic resolution . Scientific Data , 8 ( 1 ), 122 . 10.1038/s41597-021-00904-z 33927203 PMC8084962

[b37] Wirgin , A. ( 2004 ). The inverse crime . arXiv . 10.48550/ARXIV.MATH-PH/0401050

[b38] Wong , E. ( 2012 ). Optimized phase schedules for minimizing peak RF power in simultaneous multi-slice RF excitation pulses . ISMRM , Melbourne, Australia. https://www.researchgate.net/publication/280962870_Optimized_Phase_Schedules_for_Minimizing_Peak_RF_Power_in_Simultaneous_Multi-Slice_RF_Excitation_Pulses

[b39] Wu , W. , Koopmans , P. J. , Frost , R. , & Miller , K. L. ( 2016 ). Reducing slab boundary artifacts in three‐dimensional multislab diffusion MRI using nonlinear inversion for slab profile encoding (NPEN) . Magnetic Resonance in Medicine , 76 ( 4 ), 1183 – 1195 . 10.1002/mrm.26027 26510172 PMC4854328

[b40] Zahneisen , B. , Poser , B. A. , Ernst , T. , & Stenger , V. A. ( 2013 ). Three-dimensional Fourier encoding of simultaneously excited slices: Generalized acquisition and reconstruction framework . Magnetic Resonance in Medicine , 71 ( 6 ), 2071 – 2081 . 10.1002/mrm.24875 23878075 PMC3865111

