# Supplementary material for: Pseudo partition-encoded simultaneous multislab (pPRISM) for rapid, navigator-free submillimeter diffusion MRI with reduced slab-boundary signal loss
Source: Imaging Neurosci (Camb). 2025 Jan 3;3:imag_a_00417. doi: 10.1162/imag_a_00417 (PMC12301896; doi:10.1162/imag_a_00417)
Supplement: Supplementary Material [file imag_a_00417-supp.pdf]

Supplementary Materials

Orthogonality of PRISM encoding pattern

Here we prove the orthogonality of PRISM encoding mathematically. The description of PRISM encoding is copied below for readers' convenience.

Description of PRISM encoding

Assuming M slabs are RF-excited simultaneously and each slab is to be partitioned into n_p slices as illustrated in Figure A1. The distance between adjacent slabs is $n_d\times \Delta_z$ where Δ_z denotes the slab thickness and n_d is a positive integer. The field-of-view size along z direction (FOV_z) is $M\times n_d\times\Delta_z$. In k-space, the sampled data along k_z axis can be formulated as $\Gamma(k\Delta k_z)$ where k is of integer and $\Delta k_z=(2\pi/FOV_z)$.

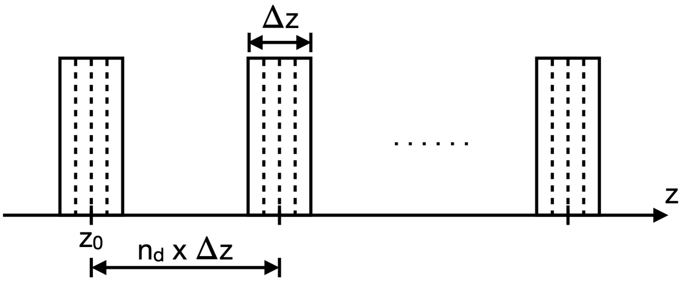

Figure A1: The 1D profile of simultaneous multislab excitation. Each slab is to be partitioned into n_p slices. The slab thickness is Δ_z and the distance between adjacent slabs is $n_d\times \Delta_z$. The total number of slabs is M.

To encode the simultaneously excited slabs orthogonally, we hypothesized that only $M\times n_p$ samples along k_z axis are required to achieve orthogonal encoding as long as the sampling pattern fulfills the following criteria:

1) $k= k_0+q\times n+M\times n_d\times b$, where n = 0, 1, …, M-1, b = 0, 1, …, n_p-1 and k_0 can be any integer,
2) gcd(q, M) = 1 where q is of positive integer. gcd (●, ∗) denotes the greatest common divisor of ● and ∗. In other words, q and M need to be coprime. The total number of data points along k_z axis needs to remain as $M\times n_p$.

Proof

After multi-slab excitation, the spatial profile can be formulated as

$\rho(x) = \sum_{m=0}^{M-1} \rho_m(z)$, where $\rho_m(z) = 0$ if $|z - (z_0 + m \cdot n_d \Delta_z)| > \Delta_z/2$. (a1)

In the k-space, the data can be formulated as

$$\Gamma(k\Delta k_z) = \int (\sum_{m=0}^{M-1} \rho_m(z)) \cdot e^{jk\Delta k_z z} dz = \sum_{m=0}^{M-1} \int_{z_0+(m\cdot n_d-0.5)\Delta_z}^{z_0+(m\cdot n_d+0.5)\Delta_z} \rho_m(z) \cdot e^{jk\Delta k_z z} dz \ . \quad \text{(a2)}$$

Assume k= $k_0+q\times n+M\times n_d\times b$, where n=0,1,…,M-1 and b=0,1,…,n_p-1. q is of positive integer and gcd(q, M)=1. k_0 can be any integer. To form orthogonal bases, any pair of spatial encoding vectors should be orthogonal. Let $k_1 = k_0+q\times n_1+M\times n_d\times b_1$, $k_2 = k_0+q\times n_2+M\times n_d\times b_2$, then $e^{jk_1\Delta k_z z}$ and $e^{jk_2\Delta k_z z}$ across the M slabs should be orthogonal.

To unify the interval of integration, we defined $z' = z - m \cdot n_d \Delta_z$,

=> $\Gamma(k_1\Delta k_z) = \sum_{m=0}^{M-1} \int_{z_0-0.5\Delta_z}^{z_0+0.5\Delta_z} \rho_m(z' + m \cdot n_d \Delta_z) \cdot e^{jk_1\Delta k_z(z'+m\cdot n_d\Delta_z)} dz'$, and

$$\Gamma(k_2\Delta k_z) = \sum_{m=0}^{M-1} \int_{z_0-0.5\Delta_z}^{z_0+0.5\Delta_z} \rho_m(z' + m \cdot n_d \Delta_z) \cdot e^{jk_2\Delta k_z(z'+m\cdot n_d\Delta_z)} dz' \ . \quad \text{(a3)}$$

The $e^{jk_1\Delta k_z(z'+m\cdot n_d\Delta_z)}$ and $e^{jk_2\Delta k_z(z'+m\cdot n_d\Delta_z)}$ are the spatial encoding bases over the multiple slabs. The orthogonality should be examined by calculating the inner product of the two bases. Therefore,

$$\sum_{m=0}^{M-1} \int_{z_0-0.5\Delta_z}^{z_0+0.5\Delta_z} e^{jk_1\Delta k_z(z'+m\cdot n_d\Delta_z)} \cdot e^{-jk_2\Delta k_z(z'+m\cdot n_d\Delta_z)} dz'$$

$$= \sum_{m=0}^{M-1} \int_{z_0-0.5\Delta_z}^{z_0+0.5\Delta_z} e^{j(k_1-k_2)\Delta k_z z'} e^{j(k_1-k_2)\Delta k_z\cdot m\cdot n_d\Delta_z} dz'$$

$$= \int_{z_0-0.5\Delta_z}^{z_0+0.5\Delta_z} \sum_{m=0}^{M-1} e^{j(k_1-k_2)\Delta k_z z'} e^{j(k_1-k_2)\Delta k_z\cdot m\cdot n_d\Delta_z} dz'$$

$$= \int_{z_0-0.5\Delta_z}^{z_0+0.5\Delta_z} e^{j(k_1-k_2)\Delta k_z z'} \sum_{m=0}^{M-1} e^{j(k_1-k_2)\Delta k_z\cdot m\cdot n_d\Delta_z} dz'$$

$$= \sum_{m=0}^{M-1} e^{j(k_1-k_2)\Delta k_z\cdot m\cdot n_d\Delta_z} \cdot \int_{z_0-0.5\Delta_z}^{z_0+0.5\Delta_z} e^{j(k_1-k_2)\Delta k_z z'} dz' \ . \quad \text{(a4)}$$

The equation of inner product can be separated into two parts:

$$\sum_{m=0}^{M-1} e^{j(k_1-k_2)\Delta k_z\cdot m\cdot n_d\Delta_z} = \sum_{m=0}^{M-1} e^{j[q(n_1-n_2)+M\cdot n_d\cdot(b_1-b_2)]\Delta k_z\cdot m\cdot n_d\Delta_z} \ , \quad \text{(a5)}$$

and $\int_{z_0-0.5\Delta_z}^{z_0+0.5\Delta_z} e^{j(k_1-k_2)\Delta k_z z'} dz' = \int_{z_0-0.5\Delta_z}^{z_0+0.5\Delta_z} e^{j[q(n_1-n_2)+M\cdot n_d\cdot(b_1-b_2)]\Delta k_z z'} dz'$. (a6)

$\because\ \Delta k_z = \frac{2\pi}{FOV_z} = \frac{2\pi}{M \cdot n_d \Delta_z}$,

$\Rightarrow \sum_{m=0}^{M-1} e^{j[q(n_1-n_2)+M\cdot n_d\cdot(b_1-b_2)]\Delta k_z \cdot m \cdot n_d \Delta_z} = \sum_{m=0}^{M-1} e^{j[q(n_1-n_2)+M\cdot n_d\cdot(b_1-b_2)]\cdot\frac{2\pi\cdot m}{M}}$, (a7)

and $\int_{z_0-0.5\Delta_z}^{z_0+0.5\Delta_z} e^{j[q(n_1-n_2)+M\cdot n_d\cdot(b_1-b_2)]\Delta k_z z'} dz' = \int_{z_0-0.5\Delta_z}^{z_0+0.5\Delta_z} e^{j[q(n_1-n_2)+M\cdot n_d\cdot(b_1-b_2)]\frac{2\pi}{M\cdot n_d \Delta_z} z'} dz'$

$= \int_{z_0-0.5\Delta_z}^{z_0+0.5\Delta_z} e^{j(\frac{2\pi\cdot q(n_1-n_2)}{M\cdot n_d \Delta_z}+\frac{2\pi(b_1-b_2)}{\Delta_z})z'} dz'$. (a8)

If $n_1 = n_2$, then $b_1 \neq b_2$,

$\int_{z_0-0.5\Delta_z}^{z_0+0.5\Delta_z} e^{j(\frac{2\pi\cdot q(n_1-n_2)}{M\cdot n_d \Delta_z}+\frac{2\pi(b_1-b_2)}{\delta_z})z'} dz' = \int_{z_0-0.5\Delta_z}^{z_0+0.5\Delta_z} e^{j\frac{2\pi(b_1-b_2)}{\Delta_z}z'} dz' = 0.$ (a9)

If $n_1 \neq n_2$, then

$\sum_{m=0}^{M-1} e^{j[q(n_1-n_2)+M\cdot n_d\cdot(b_1-b_2)]\cdot\frac{2\pi\cdot m}{M}} = \sum_{m=0}^{M-1} e^{j(n_1-n_2)\cdot\frac{2\pi\cdot q\cdot m}{M}} e^{j2\pi\cdot n_d\cdot(b_1-b_2)\cdot m}$. (a10)

$\because\ e^{j2\pi\cdot n_d\cdot(b_1-b_2)\cdot m} = 1$,

$\sum_{m=0}^{M-1} e^{j(n_1-n_2)\cdot\frac{2\pi\cdot q\cdot m}{M}} e^{j2\pi\cdot n_d\cdot(b_1-b_2)\cdot m} = \sum_{m=0}^{M-1} e^{j(n_1-n_2)\cdot\frac{2\pi\cdot q\cdot m}{M}}$. (a11)

Eq. a11 is the sum of geometric series, therefore

$\sum_{m=0}^{M-1} e^{j(n_1-n_2)\cdot\frac{2\pi\cdot q\cdot m}{M}} = \frac{1-e^{j(n_1-n_2)\cdot\frac{2\pi\cdot q\cdot M}{M}}}{1-e^{j(n_1-n_2)\cdot\frac{2\pi\cdot q}{M}}} = \frac{1-e^{j(n_1-n_2)\cdot 2\pi\cdot q}}{1-e^{j(n_1-n_2)\cdot\frac{2\pi\cdot q}{M}}}$. (a12)

$\because$ gcd (q, M)=1, $\left(1-e^{j(n_1-n_2)\cdot\frac{2\pi\cdot q}{M}}\right) \neq 0$ for all possible non-zero values of (n_1-n_2).

Then $\frac{1-e^{j(n_1-n_2)\cdot 2\pi\cdot q}}{1-e^{j(n_1-n_2)\cdot\frac{2\pi\cdot q}{M}}} = 0$ because $(n_1-n_2)\cdot q$ is an integer. (a13)

Taken Eq. a9 and a13 together, the inner product of any pair of spatial encoding bases across multi-slabs is always 0 as long as the sampling criteria are fulfilled. Hence, the spatial patterns formed by the sampling criteria constitute an orthogonal basis.
